# Longitudinal analysis of sentiment and emotion in news media headlines using automated labelling with Transformer language models

**DOI:** 10.1371/journal.pone.0276367

**Published:** 2022-10-18

**Authors:** David Rozado, Ruth Hughes, Jamin Halberstadt

**Affiliations:** 1 Te Pūkenga–New Zealand Institute of Skills and Technology, Dunedin, Otago, New Zealand; 2 Department of Psychology, University of Otago, Dunedin, Otago, New Zealand; European Commission, ITALY

## Abstract

This work describes a chronological (2000–2019) analysis of sentiment and emotion in 23 million headlines from 47 news media outlets popular in the United States. We use Transformer language models fine-tuned for detection of sentiment (positive, negative) and Ekman’s six basic emotions (anger, disgust, fear, joy, sadness, surprise) plus neutral to automatically label the headlines. Results show an increase of sentiment negativity in headlines across written news media since the year 2000. Headlines from right-leaning news media have been, on average, consistently more negative than headlines from left-leaning outlets over the entire studied time period. The chronological analysis of headlines emotionality shows a growing proportion of headlines denoting *anger*, *fear*, *disgust* and *sadness* and a decrease in the prevalence of emotionally *neutral* headlines across the studied outlets over the 2000–2019 interval. The prevalence of headlines denoting *anger* appears to be higher, on average, in right-leaning news outlets than in left-leaning news media.

## Introduction

Headlines from written news media constitute an important source of information about current affairs. News and opinion articles headlines often establish the first point of contact between an article and potential readers, with the reader often deciding whether to engage more in-depth with an article’s content after evaluating its headline [1]. In doing so, headlines also set the tone about the main text body of the article and affect readers’ processing of articles’ content to the point of constraining further information processing and biasing readers towards specific interpretations of the article [2, 3].

The sentiment and emotionality of text has been shown to influence its virality [4]. Textual content that evokes high arousal, such as text conveying an emotion of *anger*, diffuses more profusely through online platforms [5, 6]. Emotionally charged fake news also spread further and fastest through social media [7]. A study measuring the reach of tweets found that each moral or emotional word used in a tweet increased its virality by 20 percent, on average [8]. Thus, user engagement can be maximized by news articles posts that trigger negative sentiment/emotions [9]. This creates a financial incentive for news outlets to maximize incoming web traffic by modulating the emotional saliency of headlines.

News content has also been shown to be predictive of public mood [10], public opinion [11] and outlets’ biases [12, 13]. Thus, studying the sentiment (positive/negative) and emotional payload (anger, disgust, fear, joy, sadness, surprise or neutral) of news articles headlines is of sociological interest. As far as we can tell however, a comprehensive longitudinal analysis of news media headlines sentiment and emotion remains lacking in the existing literature. Here, we attempt to remedy this knowledge gap by documenting chronologically the sentiment and emotion of headlines in a representative sample of news media outlets.

Examining written sources using human coders has been useful in the sociological analysis of text content [14–16]. Unfortunately, this approach is limited by its inability to scale to large corpora and by low intercoder reliability when examining subtle themes. Computational content analysis techniques circumvent some of the limitations of content analysis using human raters by permitting the quantification of textual attributes in vast text corpora [17, 18].

Modern machine learning language models constitute an important tool for the automated analysis of text [13, 19–21]. In particular, Transformer models [22, 23] have achieved state-of-the-art performance in numerous Natural Language Processing (NLP) tasks. A Transformer model is a deep neural network that learns words’ context and thus meaning by using a mechanism known as self-attention–a form of differentially weighting the significance of each part of the input sentence when constructing word embeddings. Transformer architectures have reached prediction accuracies that match human annotations for text classification tasks such as the labelling of sentiment polarity [23]. Thus, computational content analysis of large chronological corpora using state-of-the-art machine learning models can provide insight about the temporal dynamics of semantic content in vast textual corpora [19].

This work uses modern Transformer language models, fine-tuned for text classification, to automatically label the sentiment polarity and emotional charge of a large data set of news articles headlines (N = 23 million). The set of news outlets analyzed was derived from the AllSides Media Bias Chart 2019 v1.1 [24] which lists 47 of the most popular news media outlets in the United States. Leveraging the diachronic nature of the corpus (2000–2019), we carry out a longitudinal analysis of sentiment polarity and emotional payload over time. Using external labels of news media outlets political leanings from the AllSides organization [24], we also examine the sentiment and emotional dynamics of headlines controlling for the ideological orientation of news outlets.

## Methods

### Ethics approval

Institutional ethics approval for gathering from human raters the sentiment and emotion annotations of a subset of news media headlines was obtained from the University of Otago Ethics Committee (reference number for proposal: D21/234). The human raters recruited for the annotation of the headlines provided written informed consent to participate in the study.

### Analysis scripts and data availability

The URLs sources of articles’ headlines, the Transformer models used for sentiment/emotion predictions, the sentiment and emotion labels annotations generated by the Transformer language models for each headline, the human sentiment/emotion annotations for a small subset of headlines used as ground truth to evaluate models’ performance and the analysis scripts are available in the following repository: https://doi.org/10.5281/zenodo.5144113.

### Headlines data

The set of news media outlets analysed was derived from the AllSides organization 2019 Media Bias Chart v1.1 [24]. The human ratings of outlets’ ideological leanings were also taken from this chart. The AllSides Media Bias Chart has been used previously in the literature as a representative sample of popular U.S. news media outlets and as a ground truth of news outlets ideological leanings [6, 12, 25].

In total, we analyzed 23+ Million headlines from 47 news media outlets over the period 2000–2019. Average headline length in number of characters was 58.3. Average headline length in number of tokens (i.e. unigrams) was 9.4. See S1 File for detailed histograms about these metrics.

**Fig 1:**
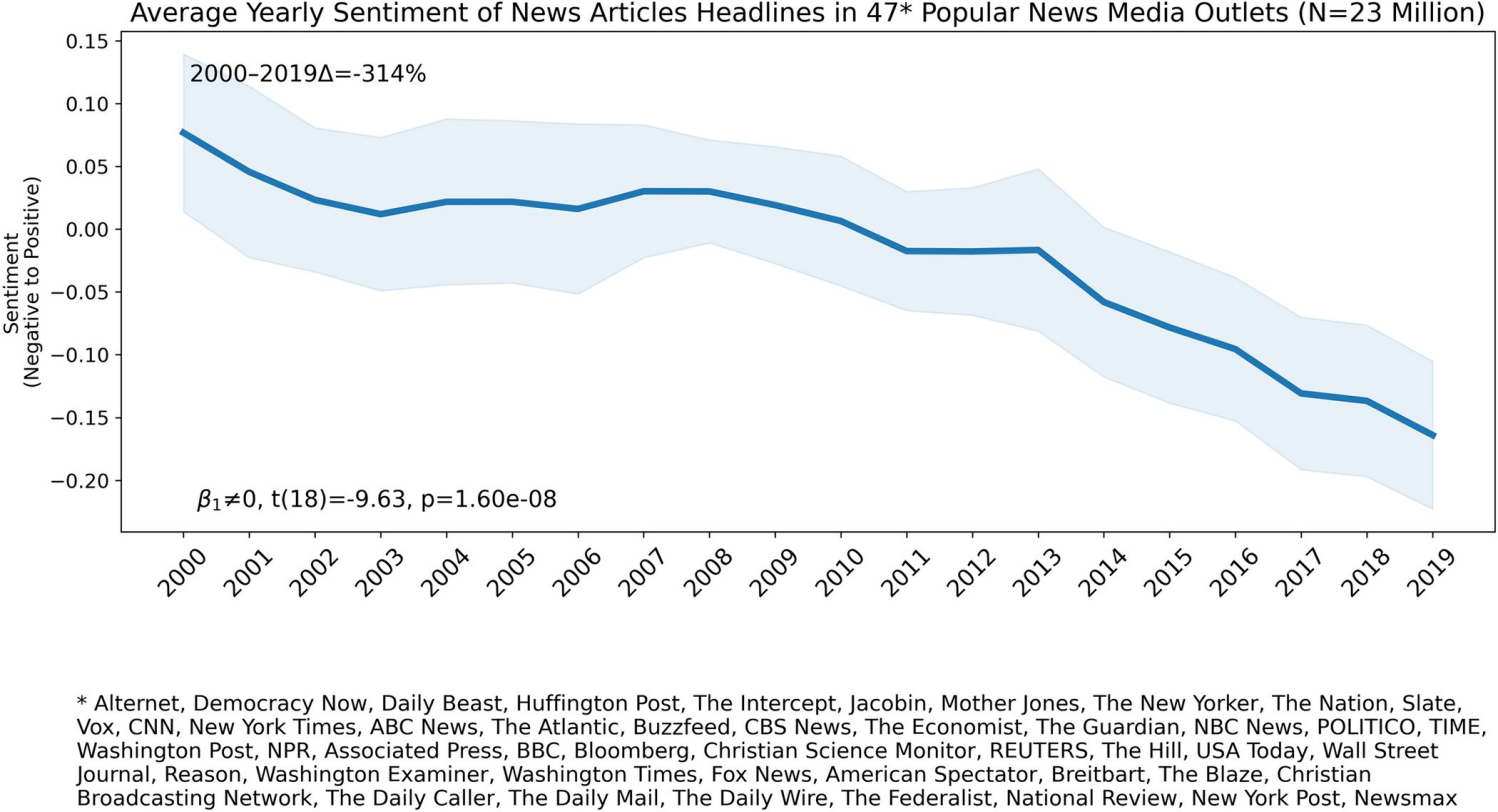
The solid blue line shows the average yearly sentiment of headlines across 47 popular news media outlets. The shaded area indicates the 95% confidence interval around the mean. A statistical test for the null hypothesis of zero slope is shown on the bottom left of the plot. The percentage change on average yearly sentiment across outlets between 2000 and 2019 is shown on the top left of the plot.

News articles headlines from the set of outlets listed in Fig 1 are available in the outlets’ online domains and/or public cache repositories such as The Internet Wayback Machine, Google cache and Common Crawl. Articles headlines were located in articles’ HTML raw data using outlet-specific XPath expressions.

To avoid unrepresentative samples, we established an inclusion criteria threshold of at least 100 outlet headlines in any given year in order for the year to be included in the outlet time series. The temporal coverage of headlines across news outlets is not uniform. For some media organizations, news articles availability in online domains or Internet cache repositories becomes sparse for earlier years. Furthermore, some news outlets popular in 2019, such as The Huffington Post or Breitbart, did not exist in the early 2000’s. Hence, our data set is sparser in headlines sample size and representativeness for earlier years in the 2000–2019 range. Nevertheless, 18 outlets in our data set have chronologically continuous availability of headlines fulfilling our inclusion criteria since the year 2000. This smaller subset with a total of 12.5 Million headlines was used to replicate our experiments and confirm the validity of the results when using a fixed set of outlets over time, see S1 File for a detailed report about the number of headlines per outlet/year in our analysis.

### Using a Transformer language model to predict the sentiment of headlines

Automated sentiment polarity annotation refers to the usage of computational tools to predict the sentiment polarity (positive or negative) of a text instance. Although the sentiment polarity of individual instances of text can sometimes be ambiguous, and humans can occasionally disagree about the sentiment of a particular piece of text, aggregating sentiment polarity over a large set of text instances provides a robust measurement of overall sentiment in a corpus since automated individual annotations accuracy is well above chance guessing.

In recent years, Transformer models have reached state-of-the-art results for automated sentiment polarity detection in natural language text [23]. In this work we use SiEBERT, a public checkpoint of a RoBERTa-large Transformer architecture [26] previously fine-tuned and evaluated for sentiment analysis on 15 data sets from diverse text sources to enhance generalization of sentiment annotations across different types of text [27]. Due to the heterogeneity of sources used for fine-tuning, SiEBERT outperforms the accuracy of a DistilBERT-based model fine-tuned solely on the popular Stanford Sentiment Treebank 2 (SST-2) data set by more than 15 percentage points (93.2 vs. 78.1 percent) [28]. The fine-tuning hyperparameters of SiEBERT were: learning rate = 2×10^−5^, number of training epochs = 3.0, number of warmup steps = 500, weight decay = 0.01 [27, 28].

To validate the usage of the Transformer model for estimating headline sentiment, we measured the performance of the fine-tuned SiEBERT model in a random sample of 1,120 headlines from our data set that we had manually annotated for positive/negative sentiment using raters recruited through Mechanical Turk. We used these labels as ground truth to measure the performance of the SiEBERT model when predicting the sentiment of news media headlines. Only individuals over 18 years old and residents of the United States of America were allowed to take part. In total, 71 individuals (measured as independent IP addresses) took part in the headlines sentiment annotation task. The SiEBERT model fine-tuned for sentiment annotation reached an accuracy of 75% on this task. Note that human sentiment annotations intercoder agreement on the same task was 80% (Cohen’s Kappa: 0.59). These results hint at the validity of the Transformer model to, on aggregate, measure the sentiment of news media headlines on par with human annotations.

We used the SiEBERT model fine-tuned for sentiment classification to automatically annotate the sentiment of every headline in our data set. We then averaged the sentiment scores of all headlines of each news outlet in any given year to obtain time series of yearly headlines sentiment polarity for each outlet. Headlines with more than 32 tokens were truncated prior to automated annotation for GPU memory computational efficiency. To further validate our results, we replicated our experiments using the popular DistilBERT-based model fine-tuned on the SST-2 data set [29].

### Using a Transformer language model to predict the emotion of headlines

Machine learning language models can also be used to detect the emotionality of text by generating emotional categories annotations for instances of natural language text. We used a public Transformer DistilRoBERTa-base checkpoint previously fine-tuned on 6 different emotion data sets for recognizing Ekman’s 6 basic emotions (*anger*, *disgust*, *fear*, *joy*, *sadness*, and *surprise*) plus *neutral* [28, 30, 31]. The fine-tuning hyperparameters of this model were: learning rate = 5×10^−5^, number of training epochs = 3.0, number of warmup steps = 500, weight decay = 0.01 [31].

The datasets used for fine tuning represent a diverse collection of text types, such as Twitter, Reddit, student self-reports or TV dialogues. The heterogeneity of data sets used for fine tuning was intended by the original authors to enhance the generalization of emotion predictions across different types of text.

To validate the ability of the model to generate accurate emotional annotations of headlines in our data set, we used the DistilRoBERTa-base fine-tuned for emotion recognition on a random sample of 5,353 headlines from our data set that we had annotated through Mechanical Turk for Ekman’s 6 basic emotion types plus *neutral* and that we used as ground truth to estimate model’s performance. Only individuals over 18 years old and residents of the United States of America were allowed to take part. In total, 143 individuals (measured as independent IP addresses) took part in the headlines’ emotion annotation task.

The DistilRoBERTa model achieved 39% classification accuracy on the task of classifying the headlines for which we had human-generated classification labels and which we used as ground truth (random guessing would be expected to reach 14%). Note that human interrater agreement on this task was also very low, 36%. See S1 File for detailed analysis. Also, since the emotion classes are not balanced in the data set of human annotated headlines’ emotionality, the accuracy metric is not particularly informative. Thus, we report the weighted precision, recall and F-1 scores of the model as 0.37, 0.39 and 0.36 respectively, see S1 File for detailed reporting for each emotional category and corresponding confusion matrices. Cohen’s kappa between model predictions and ground truth was 0.16. Matthew’s correlation coefficient between model predictions and ground truth was 0.16. Both metrics are relatively low but above the 0 level indicative of weighted random guessing. The performance of the model was above chance guessing for all emotional categories except *surprise*. Thus, in the Results section we drop this category for all subsequent analyses.

Interrater agreement between human raters for the emotion annotation task was 36% (Cohen’s Kappa = 0.16). Thus, interrater agreement was better than chance but relatively low. This is suggestive of the emotional annotation task being inherently ambiguous and/or subjective. For all emotional categories except *surprise*, interrater agreement between pairs of humans and between humans and the model was very similar. Thus, the performance of the model is mostly on par with human annotations. When using such a model to annotate a large number of headlines aggregated by year, yearly central tendency estimations should be more robust than noisy individual headline predictions.

To confirm that the automated model can detect overall trends in the emotional valence of headlines over time, we carried out a simulation using the true positive and false positive rates of the model for the different emotion categories to generate simulated annotations of illustrative hardcoded trends (see S1 File for details), and averaging those simulated predictions per year. When averaging a small set of simulated headlines emotion predictions per year (N = 100), the resulting average is unable to capture the underlying dynamics of headline emotionality. However, when aggregating a larger set of simulated headlines emotion predictions per year (N = 2,000), the resulting average is able to loosely capture the emotional dynamics of most emotion categories. When aggregating an even larger set of simulated headlines emotion predictions per year (N = 10,000 or N = 100,000), the resulting average is able to capture the emotional dynamics of all emotion categories except *surprise* with moderate to very high correlation. The underperformance in the simulation of the *surprise* category was expected since the prediction accuracy of the model on this particular category was on par with chance guessing. Note also that our data set contains a very large number of headlines per year: a minimum of more than 300,000 for the year 2000, and more than 1 million headlines per year since 2009 (see S1 File for detailed breakdown by outlet and year). Thus, allowing yearly central tendencies to reliably determine the emotional dynamics of headlines. A word cloud of the most prevailing words in each emotional category of headlines is included as S1 File to provide further support for the accuracy of the automated annotation method.

## Results

### Chronological analysis of sentiment in news articles headlines

Fig 1 shows the average yearly sentiment of news articles headlines across the 47 popular news outlets analyzed. A pattern of increasing negative sentiment in headlines over time is apparent. A linear regression t-test to determine whether the slope of the regression line differs significantly from zero was conducted: t(18) = -9.63, p<10^−7^. The percentage change in the average sentiment of headlines from the year 2000 to the year 2019 is -314%. The slope of growing negativity appears to increase post-2010. A Chow Test [32] for structural break detection in 2010 is significant (F = 28.83, p<10–5).

A potential confound in Fig 1 is that more recent years aggregate a larger number of outlets. Thus, the pattern in Fig 1 could be due to a qualitatively different mix of outlets over time. However, redoing the analysis in Fig 1 using 12.5 million headlines from the 18 news media outlets in the data set with continuous availability of news articles headlines since the year 2000 also shows a pattern of declining sentiment in headlines; see S1 File for details.

We replicate the analysis in Fig 1 using a different Transformer model (DistilBert) fine-tuned on the SST-2 sentiment data set. This variation of the analysis produces very similar results to those reported in Fig 1; see S1 File for details.

### Sentiment of news articles headlines by ideological leanings of news outlets

**Fig 2:**
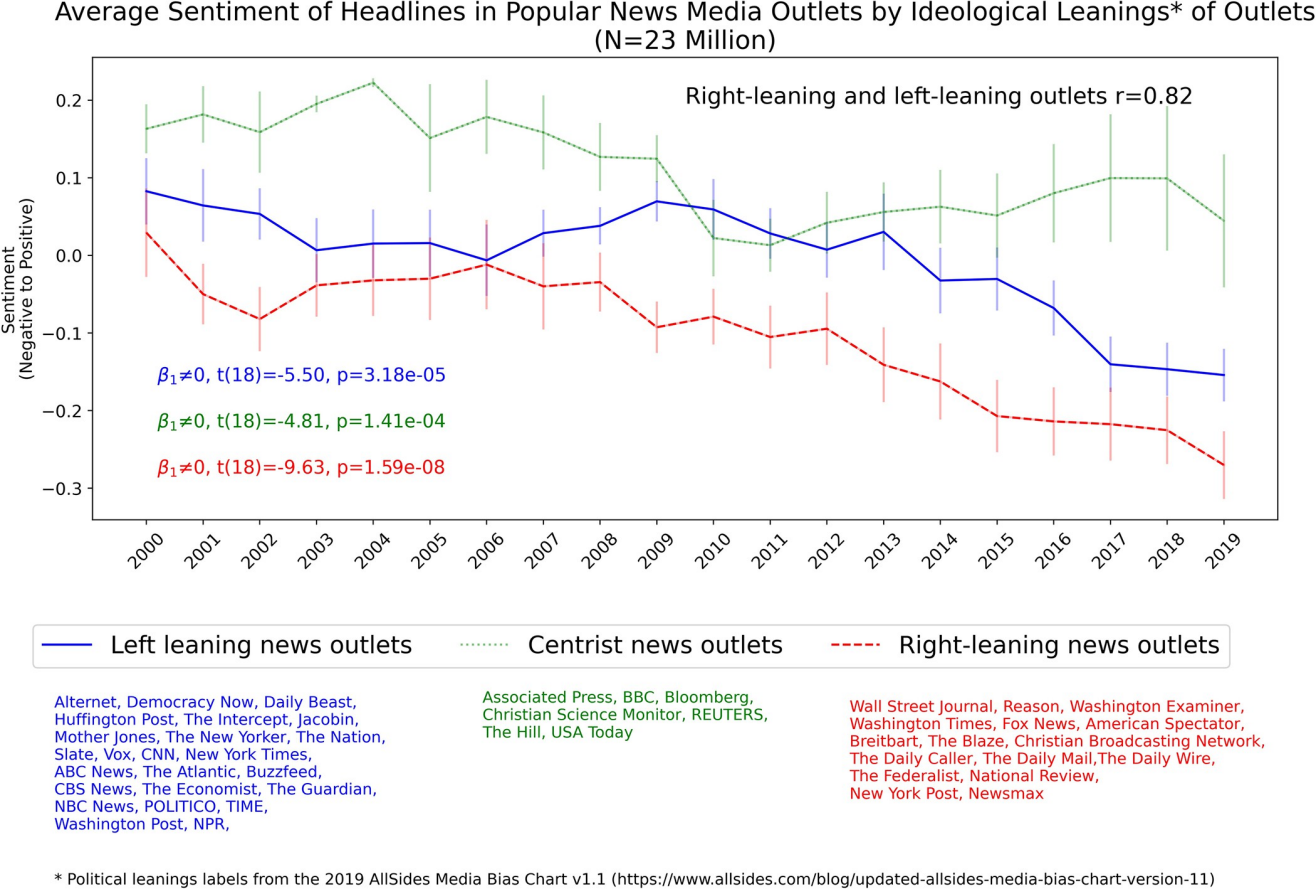
Average yearly sentiment of headlines grouped by the ideological leanings of news outlets using human ratings of outlets political bias from the 2019 AllSides Media Bias Chart v1.1 [24]. The figure displays the standard error bars of the average yearly sentiment for outlets within each color-coded political orientation category. For each ideological grouping, statistical tests for the null hypothesis of zero slope are shown on the bottom left of the plot.

Aggregating the sentiment of headlines according to the ideological leanings of news outlets, using human ratings of outlet political leanings from the 2019 AllSides Media Bias Chart v1.1 [24], shows that the pattern of increasing negativity in news headlines is consistent across left-leaning and right-leaning outlets, see Fig 2. Both right-leaning and left-leaning news outlets display increasing negative sentiment in their headlines since the year 2000. There is a high degree of correlation in the sentiment of headlines between right-leaning and left-leaning outlets (r = 0.82). On average, right-leaning news outlets have historically tended to use more negative headlines than left-leaning news outlets and continue to do so in 2019. Centrist news outlets appear to use less negative headlines than both right and left-leaning news outlets but the small set of outlets (N = 7) classified as centrists by the 2019 AllSides Media Bias Chart v1.1 warrants caution when interpreting the external validity of the centrist outlets trendline. Replicating this analysis using only the 18 media outlets with news articles headlines available since the year 2000 shows similar trends to those in Fig 2, with the caveat that the declining sentiment trend for right-leaning outlets is milder (see S1 File).

### Chronological analysis of emotion in news articles headlines

**Fig 3:**
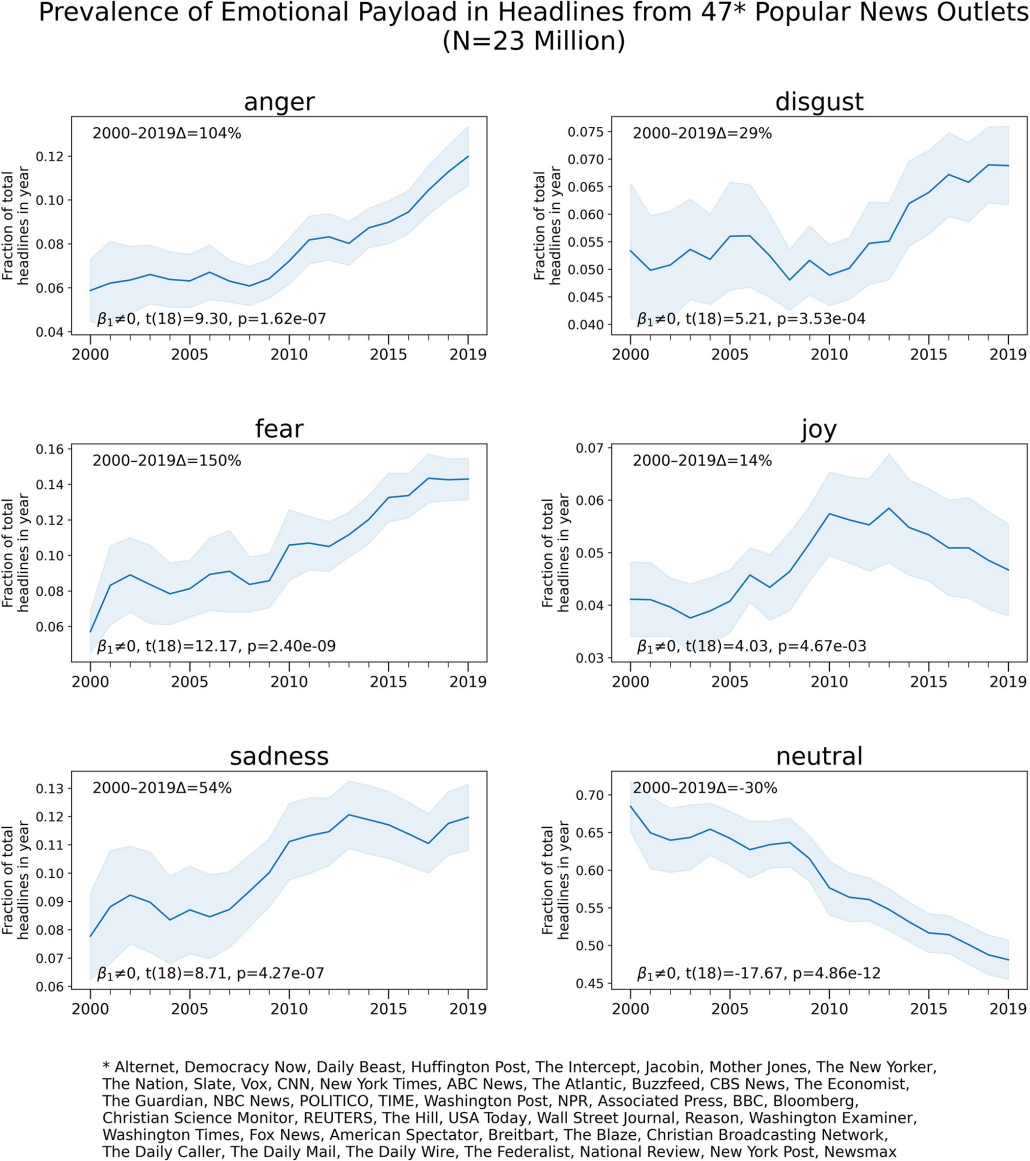
Average yearly prevalence of news articles headlines denoting different types of emotionality in 47 popular news media outlets. The shaded gray area indicates the 95% confidence interval around the mean. Note the different scale of the Y axes for the different emotion types. For each emotional category, statistical tests for the null hypothesis of zero slope are shown on the bottom left of each subplot. Reported p-values have been Bonferroni-corrected for multiple comparisons. The percentage changes between 2000 and 2019 are shown on the top left of each subplot.

Next, we analyze the emotional charge of headlines using the emotion predictions of the DistilRoBERTa-base Transformer model fine-tuned for emotion labelling. The aggregation of average yearly prevalence of emotional labels across the 47 popular news outlets analyzed is shown in Fig 3. Linear regression t-tests to determine whether the slope of the regression line differs significantly from zero were conducted for each emotion (See Fig 3 for each test’s results). Reported p-values have been Bonferroni-corrected for multiple comparisons.

An increase of 104% in the prevalence of headlines denoting *anger* since the year 2000 is apparent in Fig 3. There are also substantial increases in the prevalence of headlines denoting *fear* (+150%), *disgust* (29%) and *sadness* (+54%) in the 2000–2019 studied time range. In contrast, the prevalence of headlines with *neutral* emotion has experienced a continuous decrease (-30%) since the year 2000. The *joy* emotional category shows a curvilinear pattern with increasing proportion of headlines denoting *joy* from 2000 to 2010 and a decreasing trend from 2010 to 2019. Chow Tests [32] (Bonferroni corrected for multiple comparisons) for structural break detection in 2010 are significant for *anger* (F = 29.07, p<10^−4^), *disgust* (F = 27.97, p<10^−4^), *joy* (F = 23.69, p<10^−4^), *sadness* (F = 6.48, p<0.05) and *neutral* (F = 7.64, p<0.05). Notice the different scale of the Y-axes for the different emotion types that might exaggerate the apparent temporal dynamics of emotion categories with low prevalence such as *disgust*. To confirm that the patterns shown in Fig 3 are not the result of a different qualitative composition of outlets between the year 2000 and the year 2019, we replicate the experiment using only the 18 outlets in the data set with continuous online availability of headlines since the year 2000 (N = 12.5 million). Results show very similar trends to those displayed in Fig 3, see S1 File. Replicating the previous analysis with the 12 news outlets with more than 2,000 headlines per year since 2000 (N = 12 million), shows very similar trends. Another replication with the six news outlets with more than 10,000 headlines per year since 2000 (N = 8 million), shows very similar results to those reported in Fig 3 (see S1 File for details).

### Emotionality of news articles headlines by ideological leanings of news outlets

**Fig 4:**
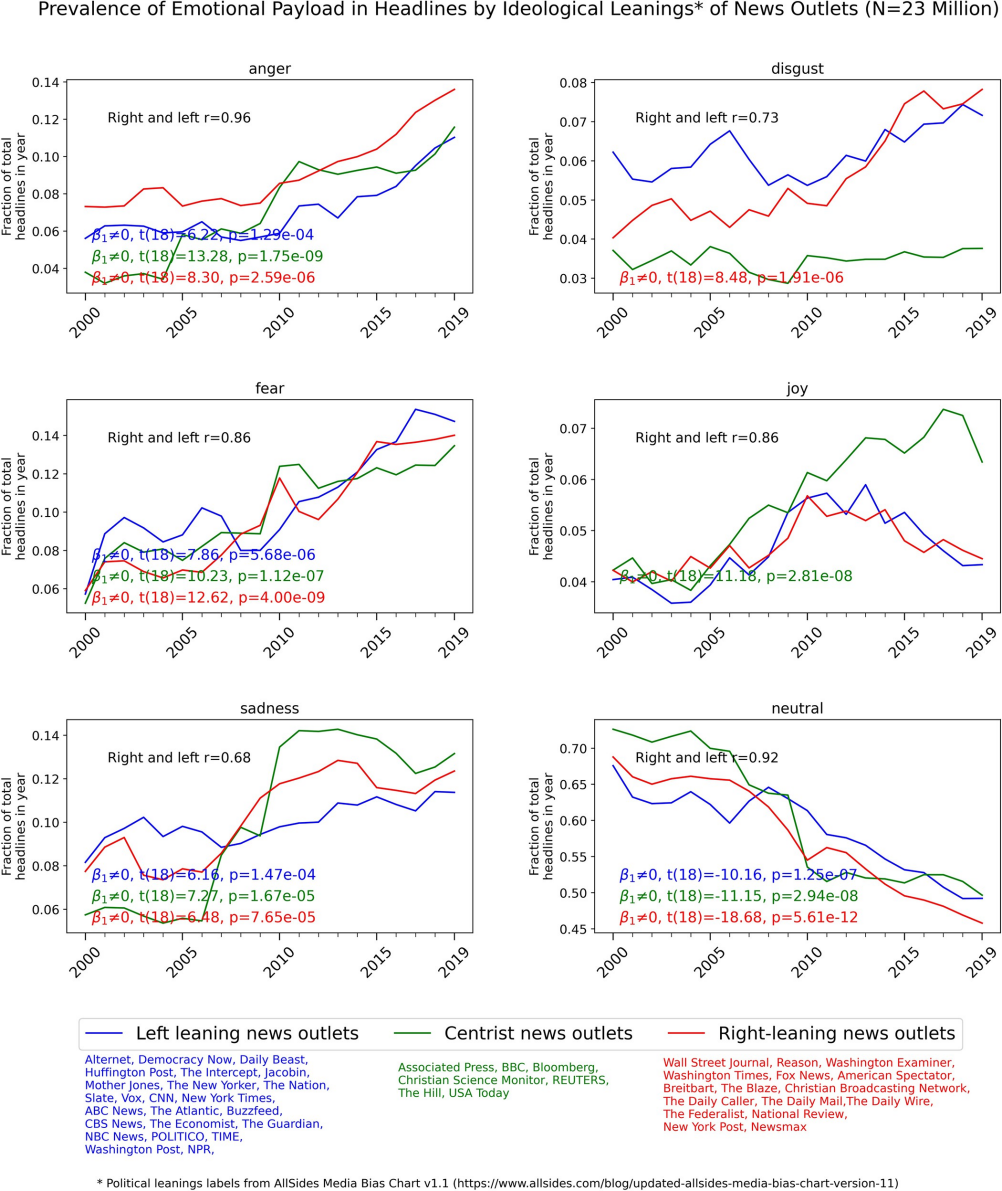
Yearly prevalence of headlines denoting different types of emotionality in 47 popular news outlets grouped by human ratings of news media ideological leanings from the 2019 AllSides Media Bias Chart v1.1 [24]. Note the different scale of the Y axes for the different emotion types. Only statistical tests within each ideological grouping for which the null hypothesis of zero slope was rejected (after Bonferroni correction for multiple comparisons) are shown on the bottom left of each plot.

Aggregating the emotionality of headlines according to the ideological leanings of the outlets, using political bias ratings from the 2019 AllSides Media Bias Chart v1.1 [24], shows that the increasing prevalence of headlines denoting *anger* is apparent in both right-leaning and left-leaning news outlets, see Fig 4. Centrist news outlets follow a similar trend over the studied time frame. *Anger* denoting headlines appear more prevalent in right-leaning outlets than in left-leaning outlets over the entire studied time period. *Fear* and *sadness* denoting headlines are also increasing across the entire ideological spectrum. The decreasing prevalence of headlines with *neutral* emotional valence appears to be consistent in left, centrist and right-leaning outlets. The degree of correlation between the emotionality of headlines in left-leaning and right-leaning news outlets is substantial for most emotion types. Replicating this analysis using only the 18 news outlets with headlines available since the year 2000 shows similar trends; see S1 File for details.

## Discussion

The results of this work show an increase of sentiment negativity in headlines across news media outlets popular in the United States since at least the year 2000. The sentiment of headlines in right-leaning news outlets has been, on average, more negative than the sentiment of headlines in left-leaning news outlets for the entirety of the 2000–2019 studied time interval. Also, since at least the year 2008, there has been a substantial increase in the prevalence of headlines denoting *anger* across popular news media outlets. Here as well, right-leaning news media appear, on average, to have used a higher proportion of *anger* denoting headlines than left-leaning news outlets. The prevalence of headlines denoting *fear* and *sadness* has also increased overall during the 2000–2019 interval. Within the same temporal period, the proportion of headlines with *neutral* emotional valence has markedly decreased across the entire news media ideological spectrum.

The higher prevalence of negativity and anger in right-leaning news media is noteworthy. Perhaps this is due to right-leaning news media simply using more negative language than left-leaning news media to describe the same phenomena. Alternatively, the higher negativity and anger undertones in headlines from right-leaning news media could be driven by differences in topic coverage between both types of outlets. Clarifying the underlying reasons for the different sentiment and emotional undertones of headlines between left-leaning and right-leaning news media could be an avenue for relevant future research.

The structural break in the sentiment polarity and the emotional payload of headlines around 2010 is intriguing, although the short nature of the time series under investigation (just 20 years of observations) makes the reliability uncertain. Due to the methodological limitations of our observational study, we can only speculate about its potential causes.

In the year 2009, social media giants Facebook and Twitter added the *like* and *retweet* buttons respectively to their platforms [33]. These features allowed those social media companies to collect information about how to capture users’ attention and maximize engagement through algorithmically determined personalized feeds. Information about which news articles diffused more profusely through social media percolated to news outlets by user-tracking systems such as browser cookies and social media virality metrics. In the early 2010s, media companies also began testing news media headlines across dozens of variations to determine the version that generated the highest click-through ratio [34]. Thus, a perverse incentive might have emerged in which news outlets, judging by the larger reach/popularity of their articles with negative/emotional headlines, started to drift towards increasing usage of negative sentiment/emotions in their headlines.

A limitation of this work is the frequent semantic overloading of the sentiment/emotion annotation task. The *negative* sentiment category for instance often conflates into the same umbrella notion of *negativity* text that describes suffering and/or being at the receiving end of mistreatment, as in “the Prime Minister has been a victim of defamation”, with text that denotes negative behavior or character traits, as in “the Prime Minister is selfish”. Thus, it is uncertain whether the increasing prevalence of headlines with negative connotations emphasize victimization, negative behavior/judgment or a mixture of the two.

An additional limitation of this work is the frequent ambiguity of the sentiment/emotion annotation task. The sentiment polarity and particularly the emotional payload of a text instance can be highly subjective and intercoder agreement is generally low, especially for the latter, albeit above chance guessing. For this reason, automated annotations for single instances of text can be noisy and thus unreliable. Yet, as shown in the simulation experiments (see S1 File for details), when aggregating the emotional payload over a large number of headlines, the average signal raises above the noise to provide a robust proxy of overall emotion in large text corpora. Reliable annotations at the individual headline level however would require more overdetermined emotional categories.

The imbalanced nature of the emotion labels also represents a challenge for the classification analysis. For that reason, we used performance metrics that are recommended when handling imbalanced data such as confusion matrices, precision, recall and F-1 scores. Usage of different algorithms such as decision trees are often recommended when working with imbalanced data. However, since Transformer models represent the state-of-the-art for NLP text classification, we circumscribed our analysis to their usage. Other techniques for dealing with imbalanced data such as oversampling the minority class or under sampling the majority class could have also been used. However, our relatively small number of human annotated headlines (1124 for sentiment and 5353 for emotion), constrained our ability to trim the human-annotated data set.

Another limitation of this work is the potential biases of the human raters that annotated the sentiment and emotion of news media headlines. It is conceivable that our sample of human raters, recruited through Mechanical Turk, is not representative of the general US population. For instance, the distribution of socioeconomic status among raters active in Mechanical Turk might not match the distribution of the entire US population. The impact of such potential sample bias on headlines sentiment/emotion estimation is uncertain.

A final limitation of our work is the small number of outlets falling into the centrist political orientation category according to the AllSides Media Bias Chart v1.1. Such small sample size limits the sample representativeness and constraints the external validity of the centrist outlets results reported here.

An important question raised by this work is whether the sentiment and emotionality embedded in news media headlines reflect a wider societal mood or if instead they just reflect the sentiment and emotionality prevalent or pushed by those creating news content. Financial incentives to maximize click-through ratios could be at play in increasing the sentiment polarity and emotional charge of headlines over time. Conceivably, the temptation of shaping the sentiment and emotional undertones of news headlines to advance political agendas could also be playing a role. Deciphering these unknowns is beyond the scope of this article and could be a worthy goal for future research.

To conclude, we hope this work paves the way for further exploration about the potential impact on public consciousness of growing emotionality and sentiment negativity of news media content and whether such trends are conductive to sustain public well-being. Thus, we hope that future research throws light on the potential psychological and social impact of public consumption of news media diets with increasingly negative sentiment and anger/fear/sadness undertones embedded within them.

## Supporting information

S1 File (DOCX)

## References

[1] O’Brien H. L., “Exploring user engagement in online news interactions,” *Proceedings of the American Society for Information Science and Technology*, vol. 48, no. 1, pp. 1–10, 2011, 10.1002/meet.2011.14504801088.

[2] Ecker U. K. H., Lewandowsky S., Chang E. P., and Pillai R., “The effects of subtle misinformation in news headlines,” *Journal of Experimental Psychology*: *Applied*, vol. 20, no. 4, pp. 323–335, 2014, doi: 10.1037/xap0000028 25347407

[3] Bransford J. D. and Johnson M. K., “Contextual prerequisites for understanding: Some investigations of comprehension and recall,” *Journal of Verbal Learning and Verbal Behavior*, vol. 11, no. 6, pp. 717–726, Dec. 1972, doi: 10.1016/S0022-5371(72)80006-9

[4] Hasell A., “Shared Emotion: The Social Amplification of Partisan News on Twitter,” *Digital Journalism*, vol. 9, no. 8, pp. 1085–1102, Sep. 2021, doi: 10.1080/21670811.2020.1831937

[5] Berger J. and Milkman K. L., “What Makes Online Content Viral?,” *Journal of Marketing Research*, vol. 49, no. 2, pp. 192–205, Apr. 2012, doi: 10.1509/jmr.10.0353

[6] Rathje S., Bavel J. J. V., and van der Linden S., “Out-group animosity drives engagement on social media,” *PNAS*, vol. 118, no. 26, Jun. 2021, doi: 10.1073/pnas.2024292118 34162706 PMC8256037

[7] de Souza M. P., da Silva F. R. M., Freire P. M. S., and Goldschmidt R. R., “A Linguistic-Based Method that Combines Polarity, Emotion and Grammatical Characteristics to Detect Fake News in Portuguese,” in *Proceedings of the Brazilian Symposium on Multimedia and the Web*, New York, NY, USA, Nov. 2020, pp. 217–224. doi: 10.1145/3428658.3430975

[8] Brady W. J., Wills J. A., Jost J. T., Tucker J. A., and Bavel J. J. V., “Emotion shapes the diffusion of moralized content in social networks,” *PNAS*, vol. 114, no. 28, pp. 7313–7318, Jul. 2017, doi: 10.1073/pnas.1618923114 28652356 PMC5514704

[9] Hansen L. K., Arvidsson A., Nielsen F. A., Colleoni E., and Etter M., “Good Friends, Bad News—Affect and Virality in Twitter,” in *Future Information Technology*, Berlin, Heidelberg, 2011, pp. 34–43. doi: 10.1007/978-3-642-22309-9_5

[10] Shapiro A. H., Sudhof M., and Wilson D. J., “Measuring news sentiment,” *Journal of Econometrics*, Nov. 2020, doi: 10.1016/j.jeconom.2020.07.053

[11] Rozado D., Al-Gharbi M., and Halberstadt J., “Prevalence of Prejudice-Denoting Words in News Media Discourse: A Chronological Analysis,” *Social Science Computer Review*, p. 08944393211031452, Jul. 2021, doi: 10.1177/08944393211031452

[12] Rozado D. and al-Gharbi M., “Using word embeddings to probe sentiment associations of politically loaded terms in news and opinion articles from news media outlets,” *J Comput Soc Sc*, 2021, doi: 10.1007/s42001-021-00130-y

[13] Rozado D., “Wide range screening of algorithmic bias in word embedding models using large sentiment lexicons reveals underreported bias types,” *PLOS ONE*, vol. 15, no. 4, p. e0231189, Apr. 2020, doi: 10.1371/journal.pone.0231189 32315320 PMC7173861

[14] Krippendorff K., *Content Analysis*: *An Introduction to Its Methodology*, Third edition. Los Angeles; London: SAGE Publications, Inc, 2012.

[15] Neuendorf K. A., *The Content Analysis Guidebook*, 1st edition. Thousand Oaks, Calif: SAGE Publications, Inc, 2001.

[16] Rozado D. and Atkins S., “Why Are Nondiscrimination Statements Not Diverse?,” *Acad*. *Quest*., vol. 31, no. 3, pp. 295–303, Sep. 2018, doi: 10.1007/s12129-018-9719-z

[17] Lewis S. C., Zamith R., and Hermida A., “Content Analysis in an Era of Big Data: A Hybrid Approach to Computational and Manual Methods,” *Journal of Broadcasting & Electronic Media*, vol. 57, no. 1, pp. 34–52, Jan. 2013, doi: 10.1080/08838151.2012.761702

[18] Rozado D., “Prejudice and Victimization Themes in New York Times Discourse: a Chronological Analysis,” *Acad*. *Quest*., vol. 33, no. 1, pp. 89–100, Mar. 2020, doi: 10.1007/s12129-019-09857-7

[19] Kozlowski A. C., Taddy M., and Evans J. A., “The Geometry of Culture: Analyzing the Meanings of Class through Word Embeddings,” *Am Sociol Rev*, vol. 84, no. 5, pp. 905–949, Oct. 2019, doi: 10.1177/0003122419877135

[20] S. Raza and C. Ding, “News Recommender System Considering Temporal Dynamics and News Taxonomy,” in *2019 IEEE International Conference on Big Data (Big Data)*, Dec. 2019, pp. 920–929. doi: 10.1109/BigData47090.2019.9005459

[21] S. Raza and C. Ding, “Deep Neural Network to Tradeoff between Accuracy and Diversity in a News Recommender System,” in *2021 IEEE International Conference on Big Data (Big Data)*, Dec. 2021, pp. 5246–5256. doi: 10.1109/BigData52589.2021.9671467

[22] Vaswani A. et al., “Attention is All you Need,” p. 11.

[23] Devlin J., Chang M.-W., Lee K., and Toutanova K., “BERT: Pre-training of Deep Bidirectional Transformers for Language Understanding,” 2019. doi: 10.18653/v1/N19-1423

[24] AllSides, “AllSides Media Bias Ratings,” *AllSides*, 2019. https://www.allsides.com/blog/updated-allsides-media-bias-chart-version-11 (accessed May 10, 2020).

[25] Spinde T., Kreuter C., and Gaissmaier W., “How Can the Perception of Media Bias in News Articles Be Objectively Measured?,” p. 10.

[26] Liu Y. et al., “RoBERTa: A Robustly Optimized BERT Pretraining Approach,” *arXiv*:*1907*.*11692 [cs]*, Jul. 2019, Accessed: Jul. 28, 2021. [Online]. Available: http://arxiv.org/abs/1907.11692

[27] Hartmann J., Heitmann M., Siebert C., and Schamp C., “More than a Feeling: Accuracy and Application of Sentiment Analysis,” *International Journal of Research in Marketing*, Jun. 2022, doi: 10.1016/j.ijresmar.2022.05.005

[28] Heitmann M., Siebert C., Hartmann J., and Schamp C., “More than a Feeling: Benchmarks for Sentiment Analysis Accuracy,” Social Science Research Network, Rochester, NY, SSRN Scholarly Paper ID 3489963, Jul. 2020. doi: 10.2139/ssrn.3489963

[29] Wolf T. et al., “HuggingFace’s Transformers: State-of-the-art Natural Language Processing,” *arXiv*:*1910*.*03771 [cs]*, Feb. 2020, Accessed: May 08, 2020. [Online]. Available: http://arxiv.org/abs/1910.03771

[30] Ekman P., “An argument for basic emotions,” *Cognition and Emotion*, vol. 6, no. 3–4, pp. 169–200, May 1992, doi: 10.1080/02699939208411068

[31] Hartmann J., “emotion-english-distilroberta-base.” https://huggingface.co/j-hartmann/emotion-english-distilroberta-base (accessed Jul. 28, 2021).

[32] Chow G. C., “Tests of Equality Between Sets of Coefficients in Two Linear Regressions,” *Econometrica*, vol. 28, no. 3, pp. 591–605, 1960, doi: 10.2307/1910133

[33] Haidt J. and Twenge J. M., “Opinion | This Is Our Chance to Pull Teenagers Out of the Smartphone Trap,” *The New York Times*, Jul. 31, 2021. Accessed: Aug. 18, 2021. [Online]. Available: https://www.nytimes.com/2021/07/31/opinion/smartphone-iphone-social-media-isolation.html

[34] Rose-Stockwell Tobias J. H., “The Dark Psychology of Social Networks,” *The Atlantic*, Nov. 12, 2019. https://www.theatlantic.com/magazine/archive/2019/12/social-media-democracy/600763/ (accessed Aug.20, 2021).

