# Supplementary material for: Longitudinal analysis of sentiment and emotion in news media headlines using automated labelling with Transformer language models
Source: PLoS One. 2022 Oct 18;17(10):e0276367. doi: 10.1371/journal.pone.0276367 (PMC9578611; doi:10.1371/journal.pone.0276367)
Supplement: S1 File — (DOCX) [file pone.0276367.s001.docx]

Supporting Information for Manuscript: Longitudinal Analysis of Sentiment and Emotion in News Media Headlines Using Automated Labelling with Transformer Language Models

David Rozado, Ruth Hughes, Jamin Halberstadt

**Headlines availability**

The analysis of news articles headlines from different outlets was constrained by headlines availability in outlets domains. The temporal coverage of outlet headlines fulfilling our inclusion criteria is shown in Figure S 1.


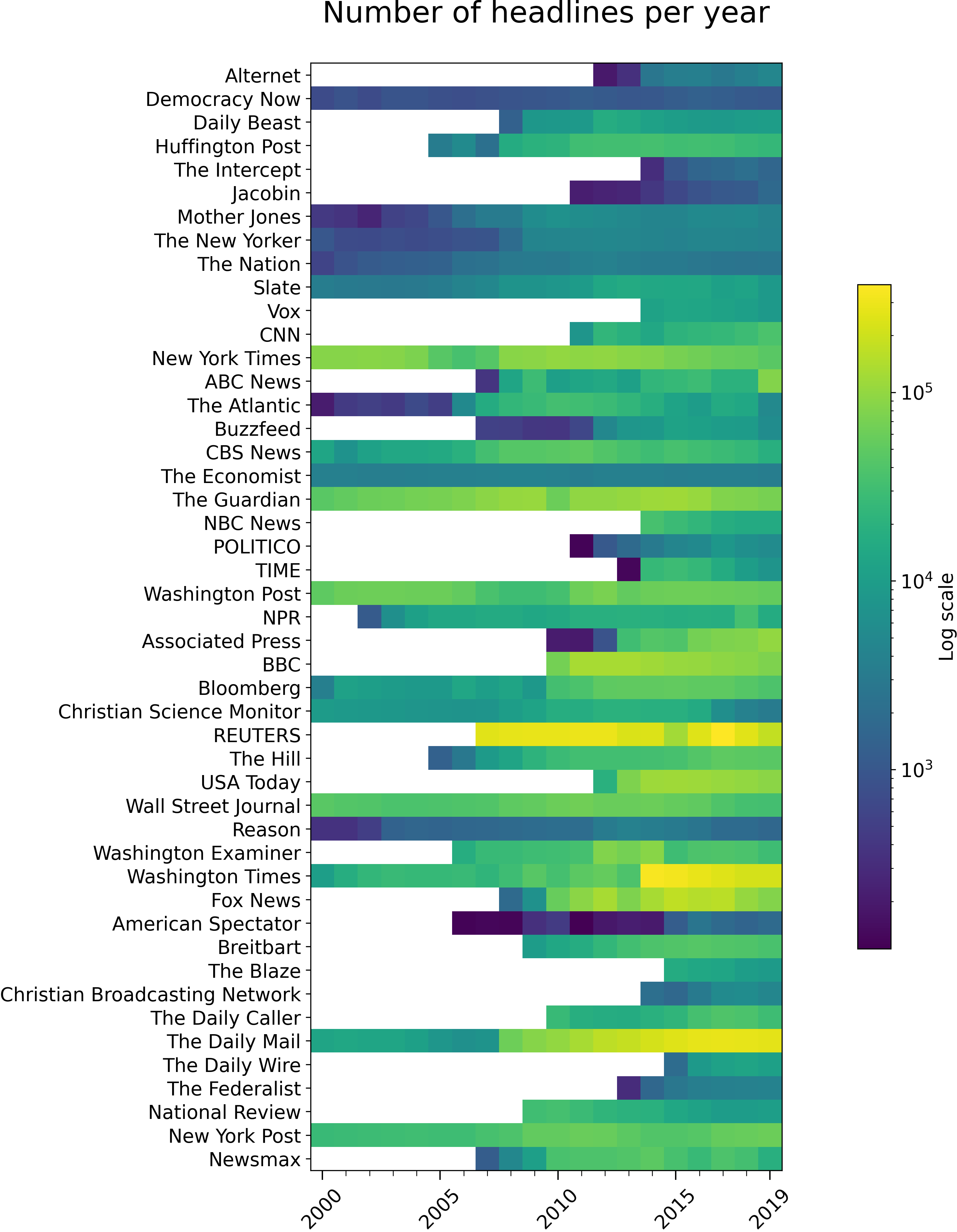


Figure S 1 Number of headlines analyzed based on their availability in news media outlets online domains

**Headlines length**

In total, we analyzed 23+ Million headlines from 47 news media outlets over the period 2000–2019. Average headline length in number of characters was 58.3. See Figure S 2 for detailed distribution.


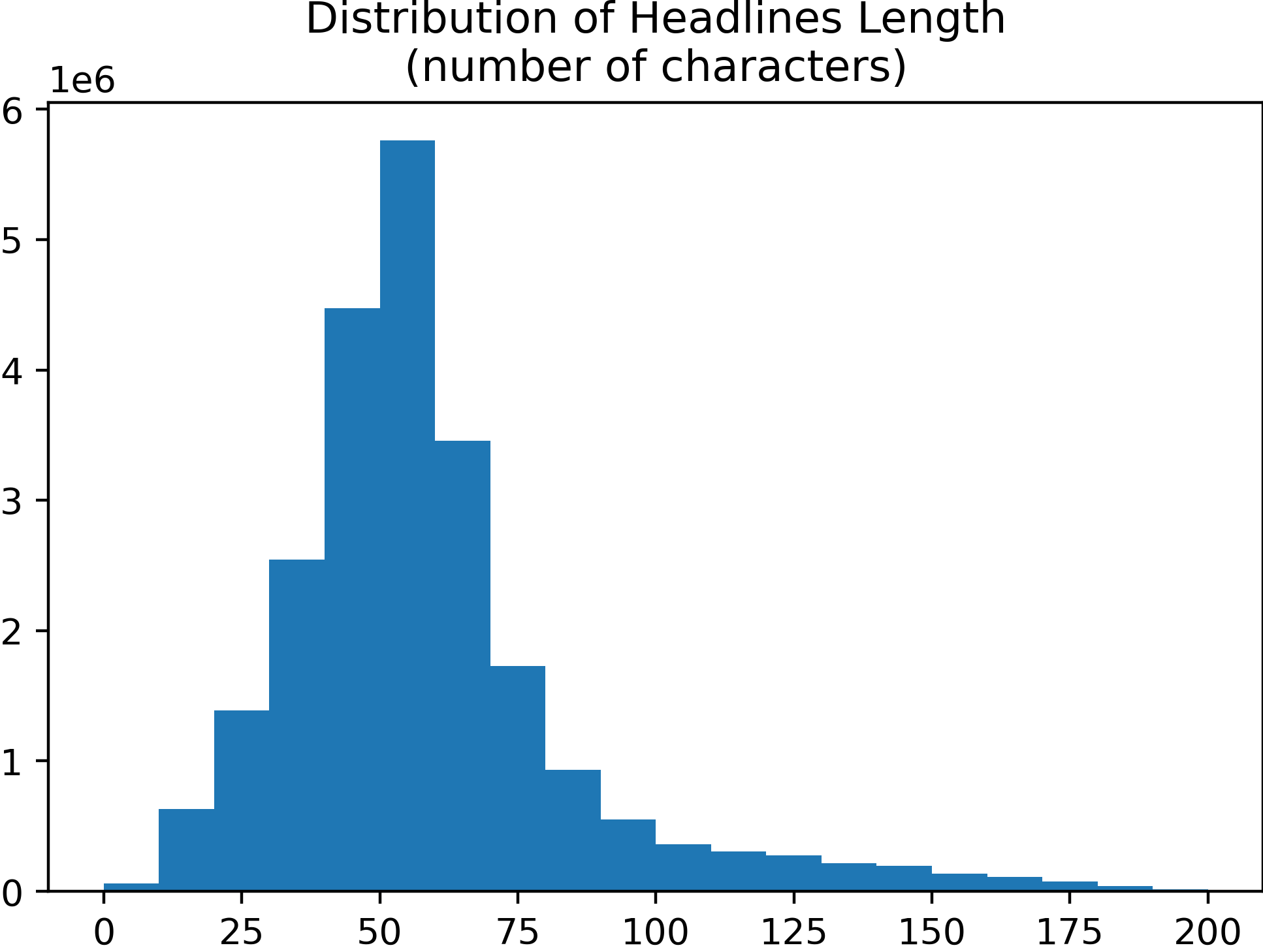


Figure S 2 Histogram of headlines length in number of characters.

Average headline length in number of tokens (i.e. unigrams) was 9.4. See Figure S 3 for detailed distribution.


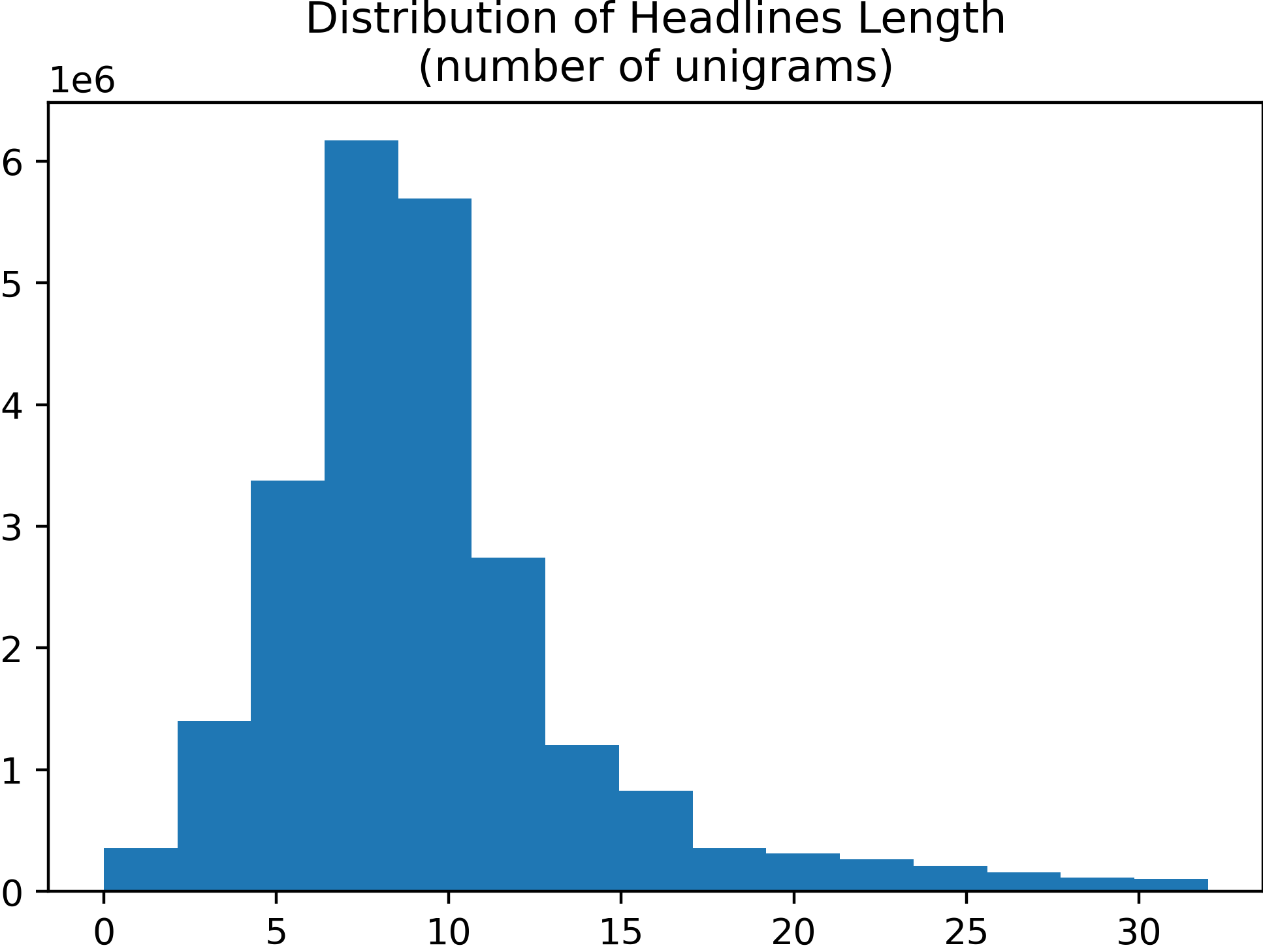


Figure S 3 Histogram of headlines length in number of unigrams.

**Validity of automated emotion labelling**

To measure the validity of the Transformer model annotations [2] for predicting the emotion of headlines, we selected a random and balanced (equal probability of an outlet being chosen) subset of headlines in our data set (N=5,353) and annotated each headline with an emotion label as judged by US English-speaking human raters recruited through Mechanical Turk. These human annotations are used as ground truth to benchmark the performance of the model against them, see Table S 1.


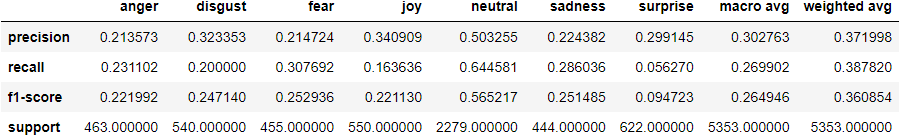


Table S 1 Classification performance of the DistilRoBERTa Transformer language model fine-tuned for emotion annotation [2] on a subset (N=5,353) of headlines in our data set annotated for emotion category ground truth by human raters.

Table S 2 shows the simulated performance metrics of weighted random guessing on the human annotated headlines for each emotional category. For all emotional categories except *surprise*, the performance of the model (see Table S 1) is better than weighted chance guessing (see Table S 2). For that reason, the *surprise* category is dropped from subsequent analysis in the main manuscript. The confusion matrix of the Transformer model for the task of emotion predictions is shown in Figure S 4.


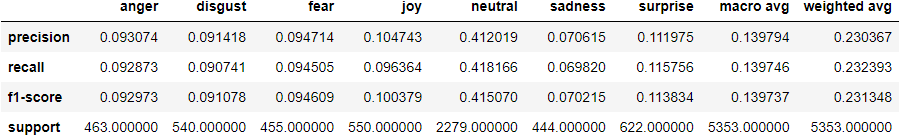


Table S 2 Classification performance of weighted random guessing on a subset (N=5,353) of headlines in our data set annotated for emotion category ground truth by human raters.


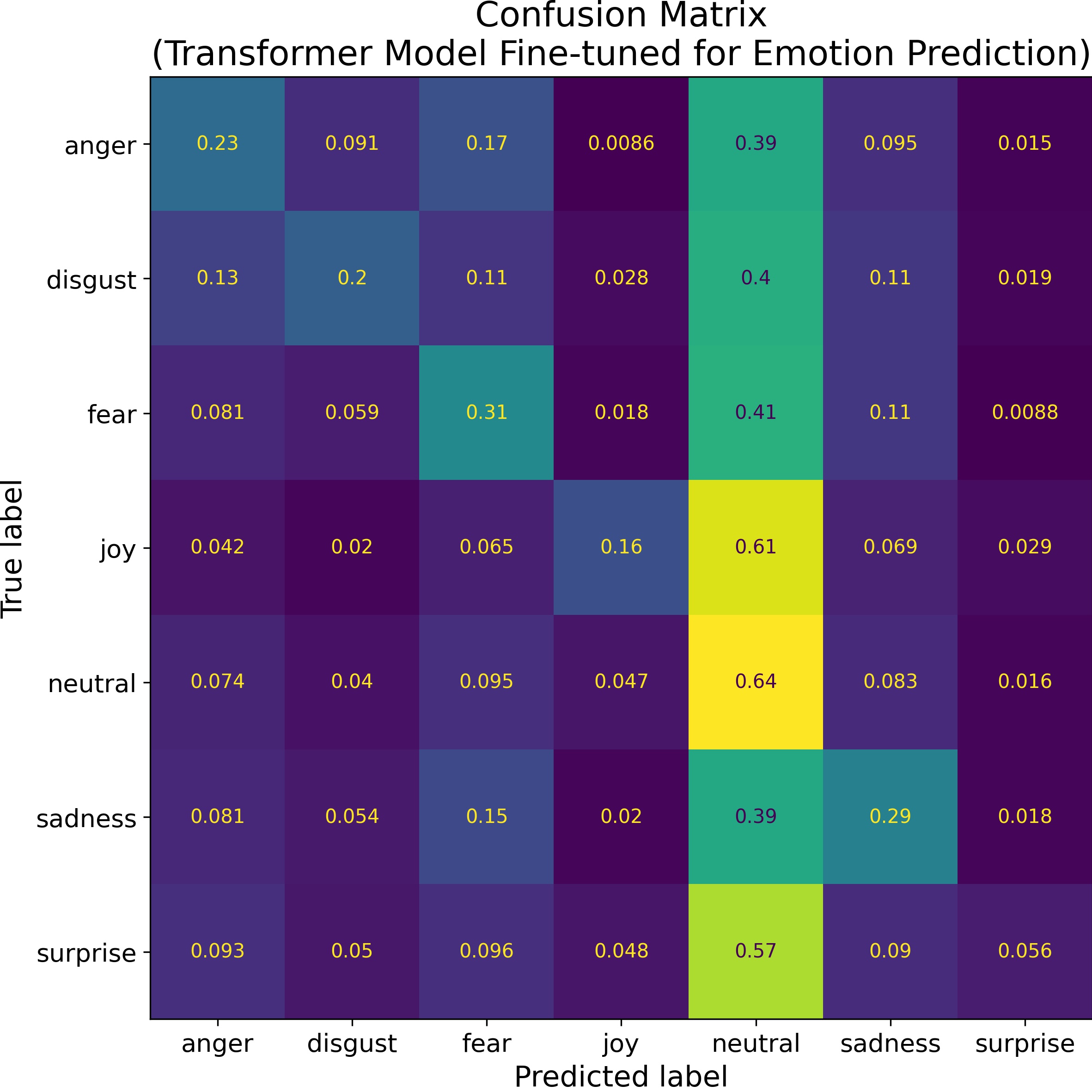


Figure S 4 The fractions in the main diagonal of the matrix denote the percentage of headlines in each emotion category correctly classified by the Transformer DistilRoBERTa-base model when using the human annotations as ground truth. Model performance is above chance guessing for all emotion categories except “surprise”.

**Interrater agreement between humans for emotion labelling**

The emotion annotation task with Ekman’s 6 basic emotions (anger, disgust, fear, joy, sadness, and surprise) plus neutral on news media headlines is inherently subjective and interrater agreement between human annotations is low. That is, often human raters disagree on whether the emotional undertones of a headline denote anger, fear, disgust or sadness for instance. In a subset of the human emotion annotations in our data set with two human ratings per headline (N=1,016), interrater agreement was 36% (Cohen’s Kappa: 0.16, Matthews correlation coefficient: 0.16), see Table S 3. Like with automated emotion labelling, these metrics are relatively low but substantially above chance guessing (compare human interrater agreement in Table S 3 with chance guessing of ground truth labels in Table S 2). Agreement between human raters was similar to agreement between human raters and the model, see Table S 3 and Table S 1. The confusion matrix between human emotion predictions is shown in Figure S 5.


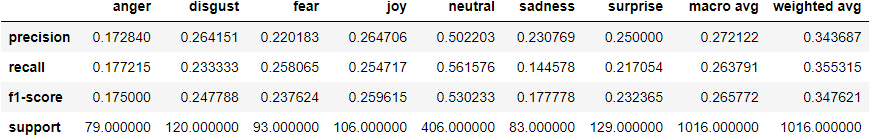


Table S 3 Human interrater agreement performance on the task of annotating the emotional category of headlines (N= 1,016) annotations. One of the human ratings is used as ground truth and the other is used as a prediction in order to compute a classification report that can be compared with automated emotion annotation.


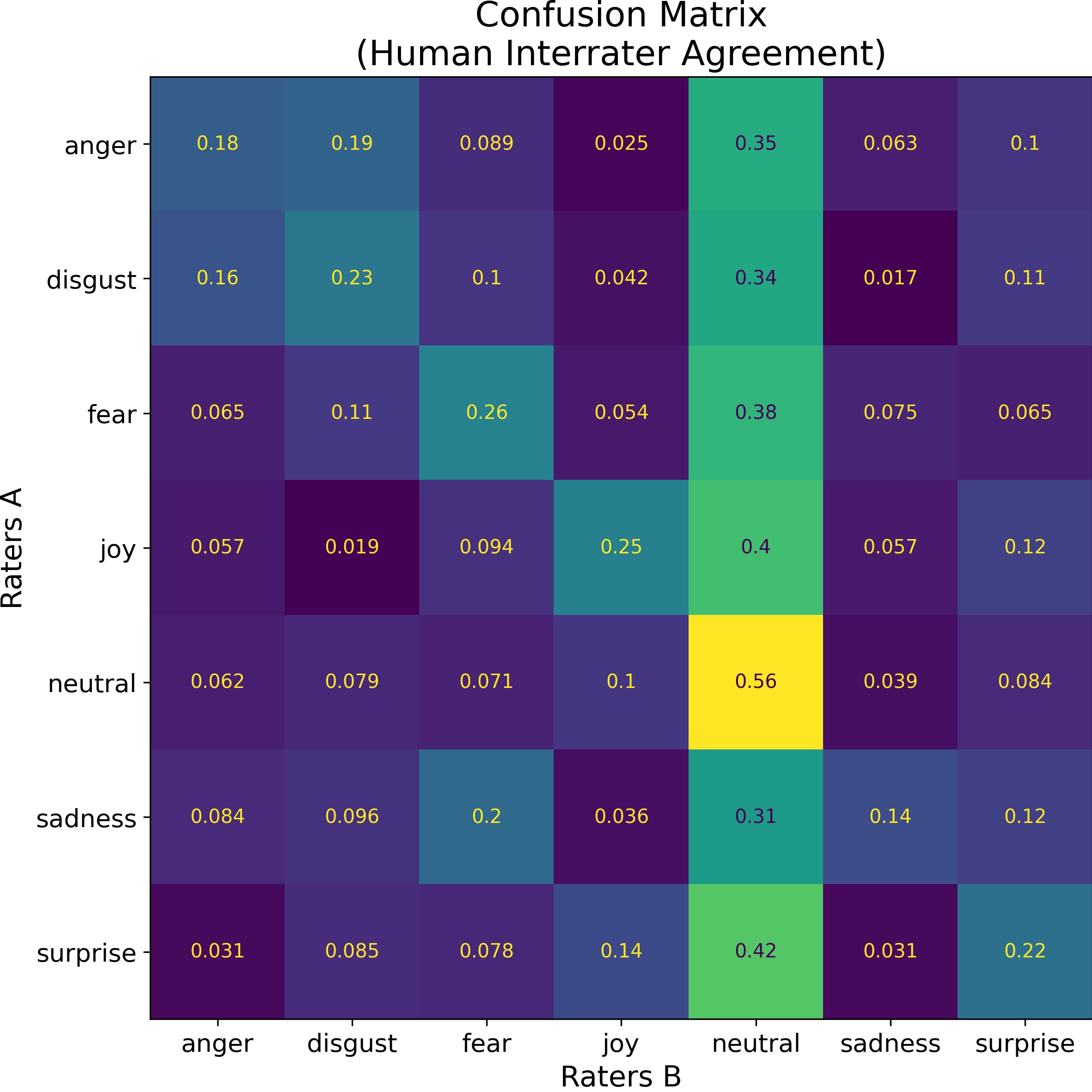


Figure S 5 The numbers in the main diagonal denote the fraction of headlines in each emotional category with the same emotion annotation between both human raters.

**Ability of the emotion annotation model with low recall and precision to detect ground truth patterns in emotional dynamics of headlines when averaging predictions over a large number of headlines**

To demonstrate that the model predictions of emotion when aggregated over a large number of headlines are able to track the emotion dynamics of headlines over time, despite its low recall and precision metrics, we carry out a simulation of emotion annotation using the actual true positive and false positive rates of the model on the 5,353 headlines annotated for emotion by humans through Mechanical Turk. We generate hardcoded ground-truth illustrative dynamics of headlines emotions using the trends contained in Figure 3 of the main manuscript. But any other trends should show the same effects described below. When the simulated predictions derived from the true positive and false positive rates of the model are averaged over a small number of headlines per year (N=100), the average of model predictions (blue continuous trend) fails to capture the underlying ground truth dynamics (dashed orange trend) for most emotion categories, see Figure S 6.


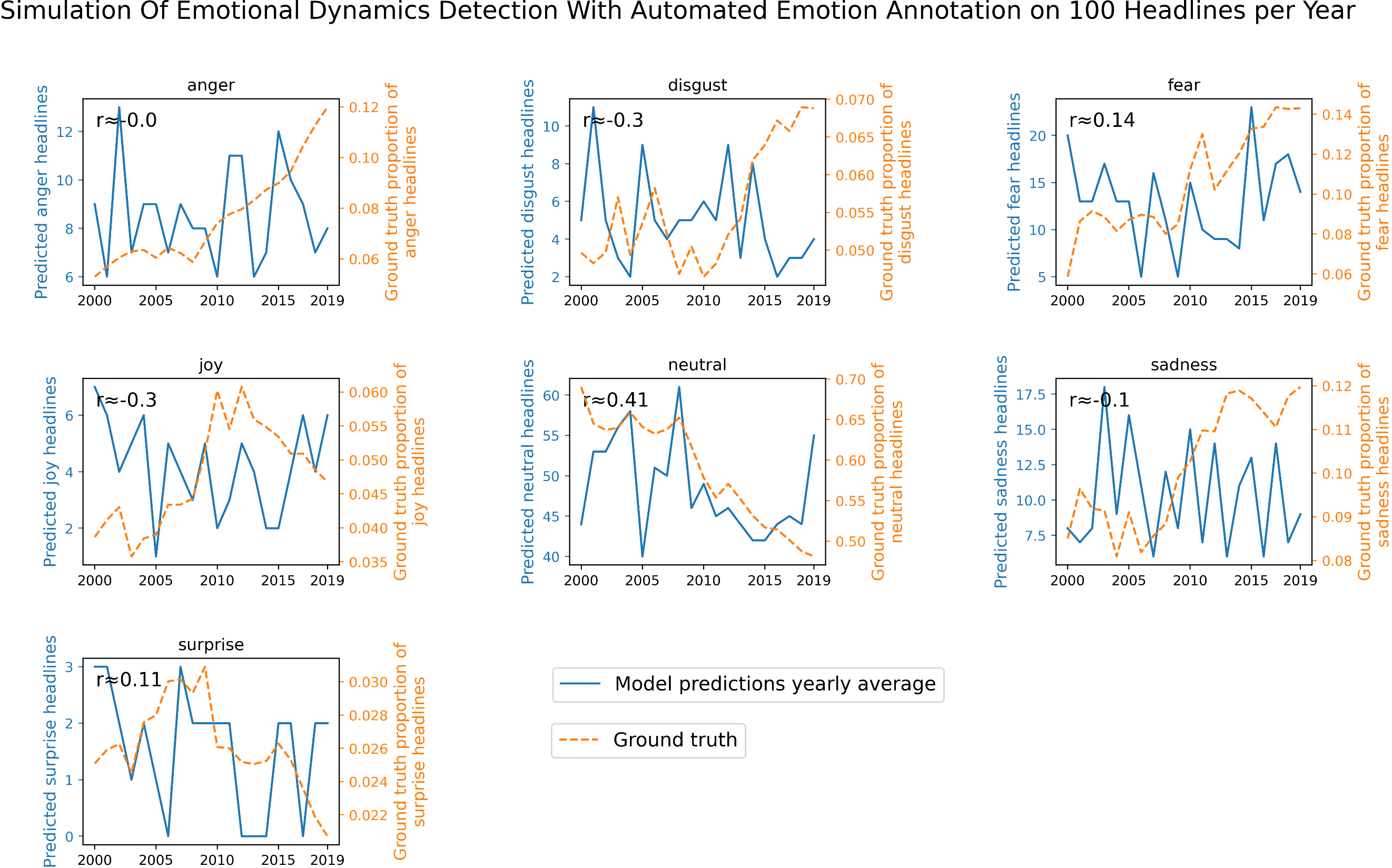


Figure S 6 Ground truth dynamics of headlines emotion (dashed orange trends) and emotion estimations (blue continuous trend) using simulated predictions with the same true positive rate and false positive rate per category as the model used in the paper for annotation of emotion categories. The blue trends above are the result of averaging 100 headlines emotion predictions per year.

In contrast, when averaging simulated predictions over a larger number of headlines per year (N=2,000), the simulated predictions averages (blue continuous trend) loosely capture the underlying pattern (dashed orange trend) for most emotional categories, see Figure S 7.


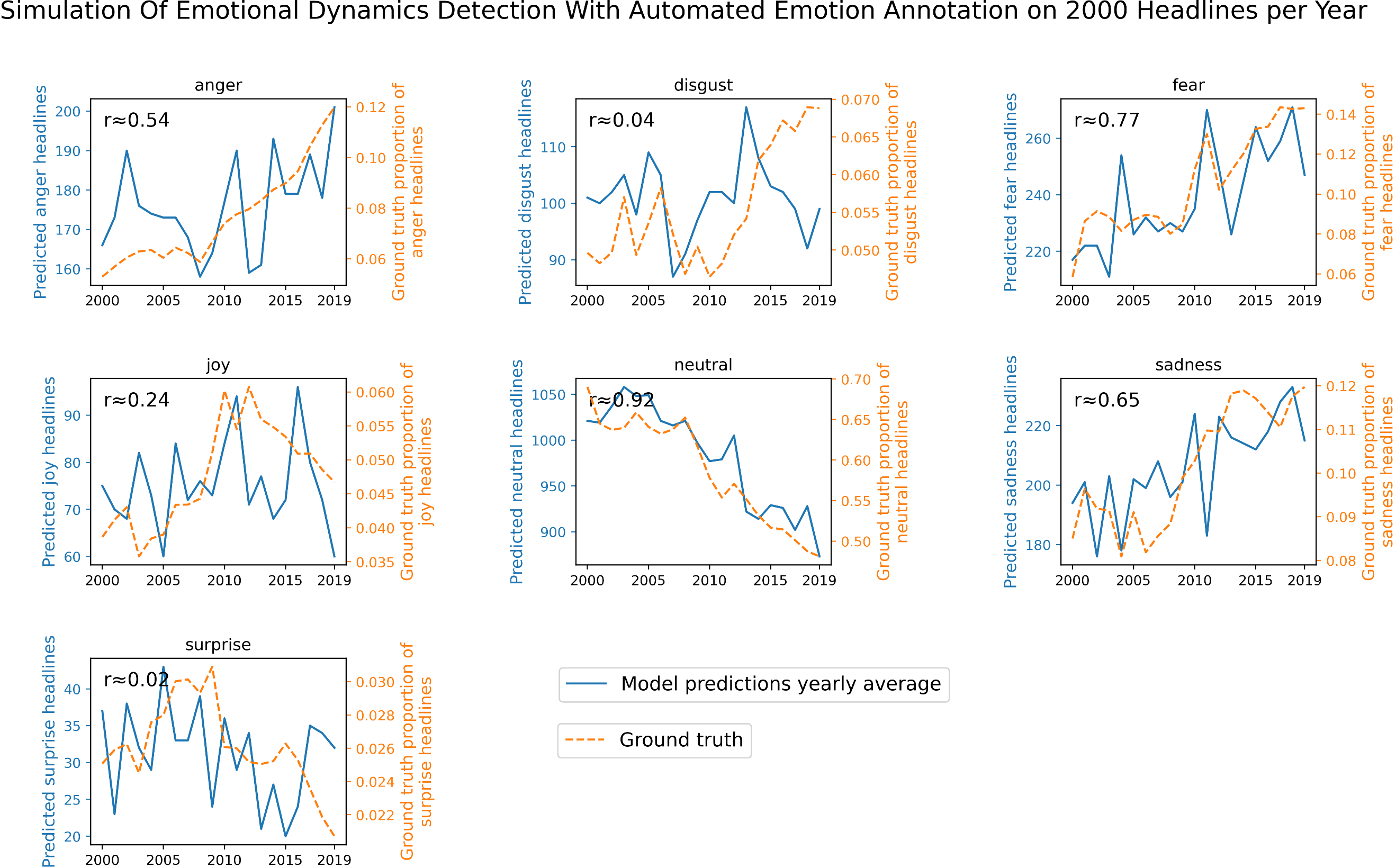


Figure S 7 Ground truth dynamics of headlines emotion (dashed orange trends) and emotion estimations (blue continuous trend) using simulated predictions with the same true positive rate and false positive rate per category as the model used in the paper for annotation of emotion categories. The blue trends above are the result of averaging 2,000 headlines emotion predictions per year.

When averaging simulated predictions over an even larger number of headlines per year (in this example N=10,000), the simulated predictions averages (blue continuous trend) capture the underlying pattern (dashed orange trend) for most emotional categories, see Figure S 8.


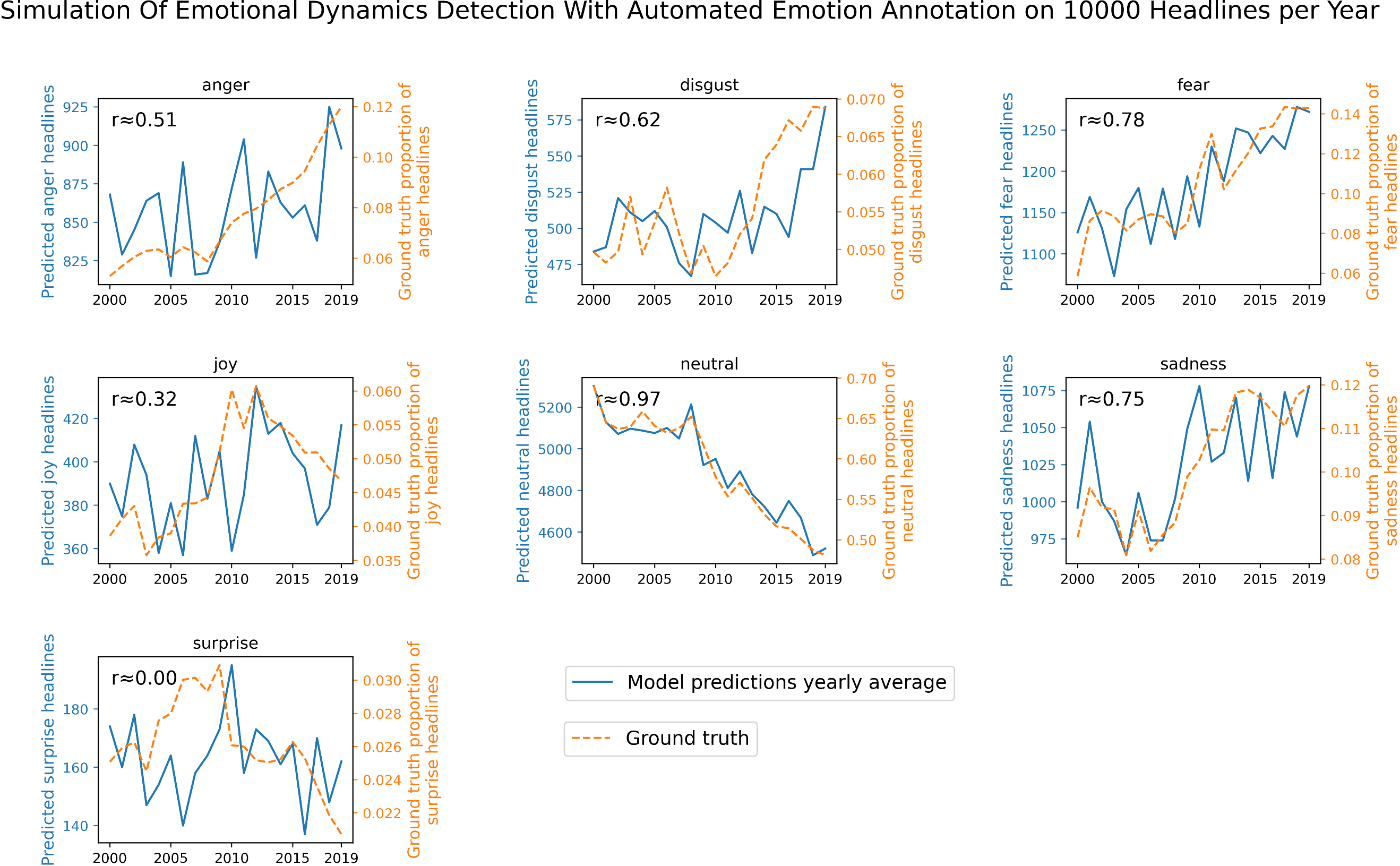


Figure S 8 Ground truth dynamics of headlines emotion (dashed orange trends) and emotion estimations (blue continuous trend) using simulated predictions with the same true positive rate and false positive rate per category as the model used in the paper for annotation of emotion categories. The blue trends above are the result of averaging 10,000 headlines emotion predictions per year.

When averaging simulated predictions over a very large number of headlines per year (N=100,000), the simulated predictions averages (blue continuous trend) precisely capture the underlying pattern (dashed orange trend), see Figure S 9, for all emotional categories except *surprise*. This is not unexpected, since model performance for this particular category was below chance guessing, see Figure S 4. We thus drop this emotional category from subsequent analysis in the main manuscript.


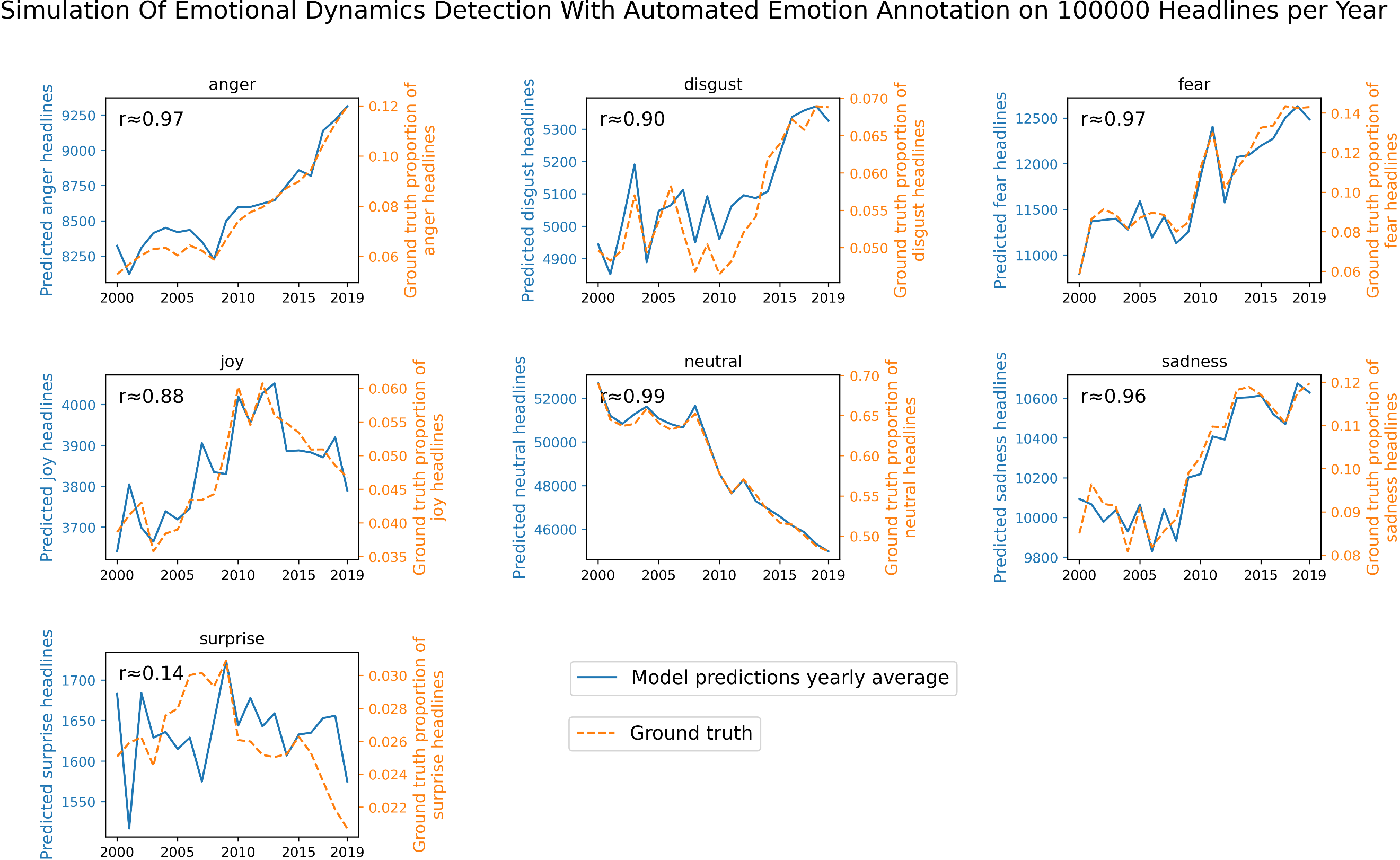


Figure S 9 Ground truth dynamics of headlines emotion (dashed orange trends) and emotion estimations (blue trend) using simulated predictions with the same true positive rate and false positive rate per category as the model used in the paper for annotation of emotion categories. The blue trends above are the result of averaging 100,000 headlines emotion predictions per year.

Notice that the number of headlines per year in our data set is always well above 100,000 headlines. The minimum number of headlines in any given year occurs for the year 2000 with over 300,000 headlines. Since 2009, the data set contains more than 1 million headlines per year, see Figure S 10.


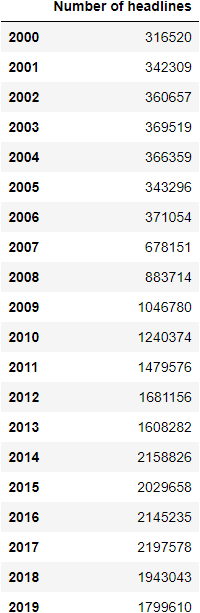


Figure S 10 Number of headlines per year in the data set of news media headlines.

**Chronological analysis of sentiment in news articles headlines** **using a fixed set of 18 news outlets with headlines availability since the year 2000**

A potential confound in Figure 1 of the main manuscript is that more recent years aggregate a larger number of outlets. Thus, the pattern in Figure 1 could be due to a qualitatively different mix of outlets over time. However, redoing the analysis in Figure 1 using 12.5 million headlines from the 18 news media outlets in the data set with continuous online availability of news articles headlines since the year 2000 also shows a pattern of declining sentiment in headlines, see Figure S 11.


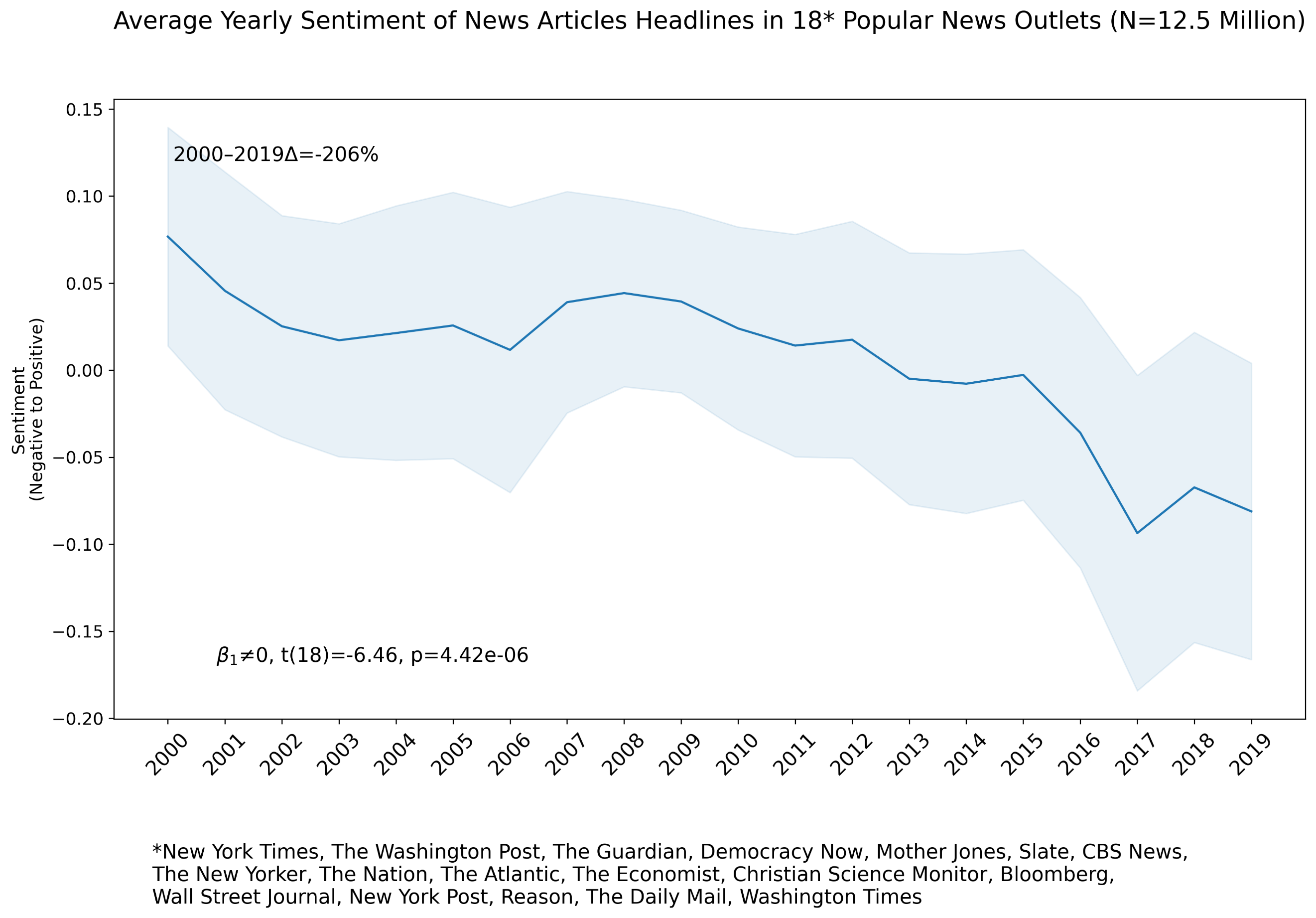


Figure S 11 The solid blue line shows the average yearly sentiment of headlines across 18 popular news media outlets. The shaded gray area indicates the 95% confidence interval around the mean. A statistical test for the null hypothesis of zero slope is shown on the bottom left of the plot. The percentage change on average yearly sentiment across outlets between 2000 and 2019 is shown on the top left of the plot.

**Chronological analysis of sentiment in news articles headlines using a different Transformer model**

Using a DistilBert Transformer model, fine-tuned on the SST-2 data set, for automated annotation of headlines’ sentiment, and averaging yearly sentiment across the 47 outlets analyzed generated similar results to those in Figure 1 of the main manuscript, see Figure S 12.


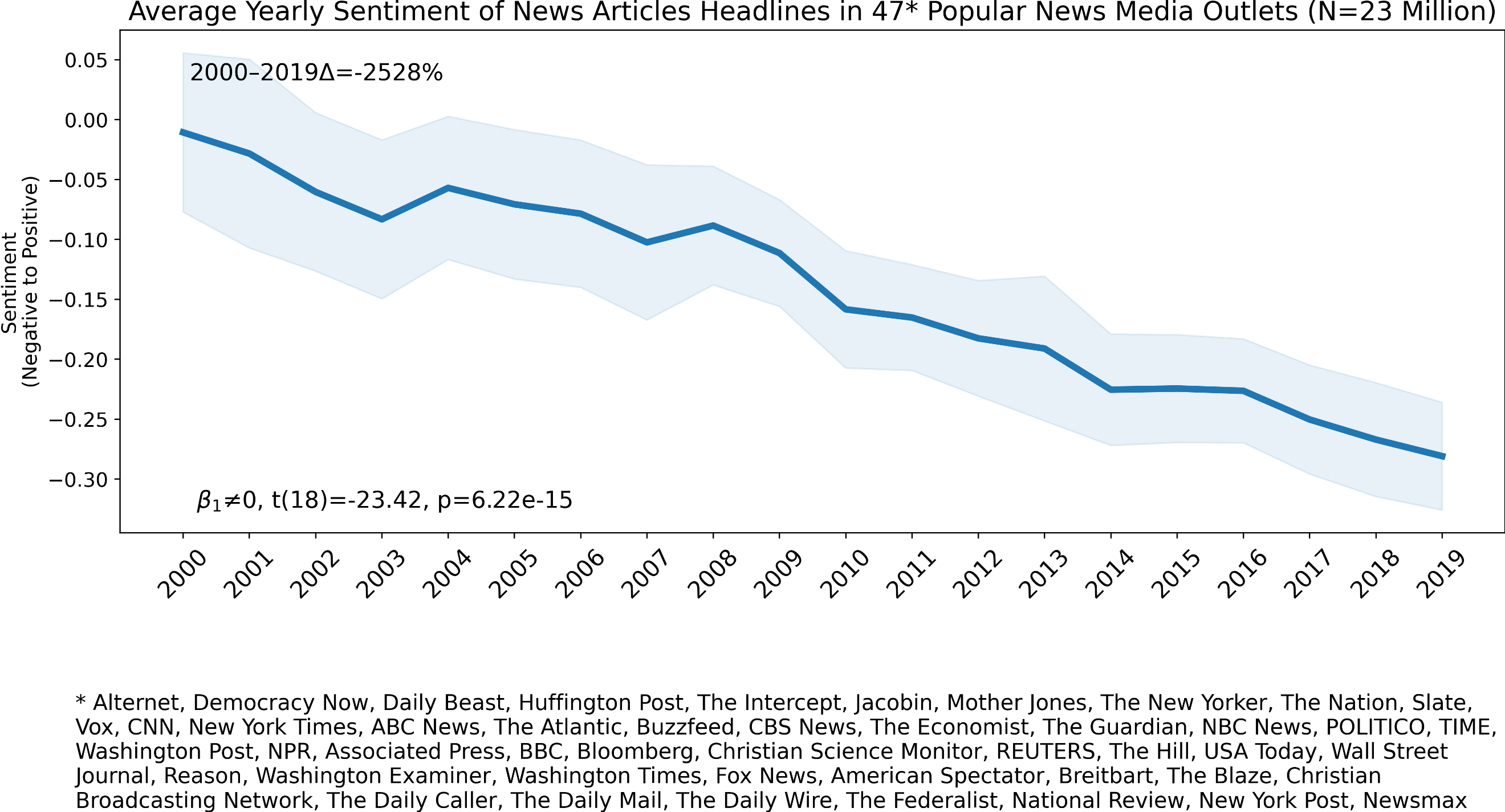


Figure S 12 The solid blue line shows the average yearly sentiment of headlines across 47 popular news media outlets using a DistilBert Transformer model fine-tuned on the SST-2 data set for sentiment annotations. The shaded gray area indicates the 95% confidence interval around the mean. A statistical test for the null hypothesis of zero slope is shown on the bottom left of the plot. The percentage change on average yearly sentiment across outlets between 2000 and 2019 is shown on the top left of the plot.

The sentiment of news articles headlines from the 18 news outlets with at least partial online availability of headlines since the year 2000 using the DistilBert Transformer model, fine-tuned on the SST-2 data set, generated the results shown in Figure S 13, which display a similar trend of declining headlines sentiment to that of Figure 1 in the main manuscript.


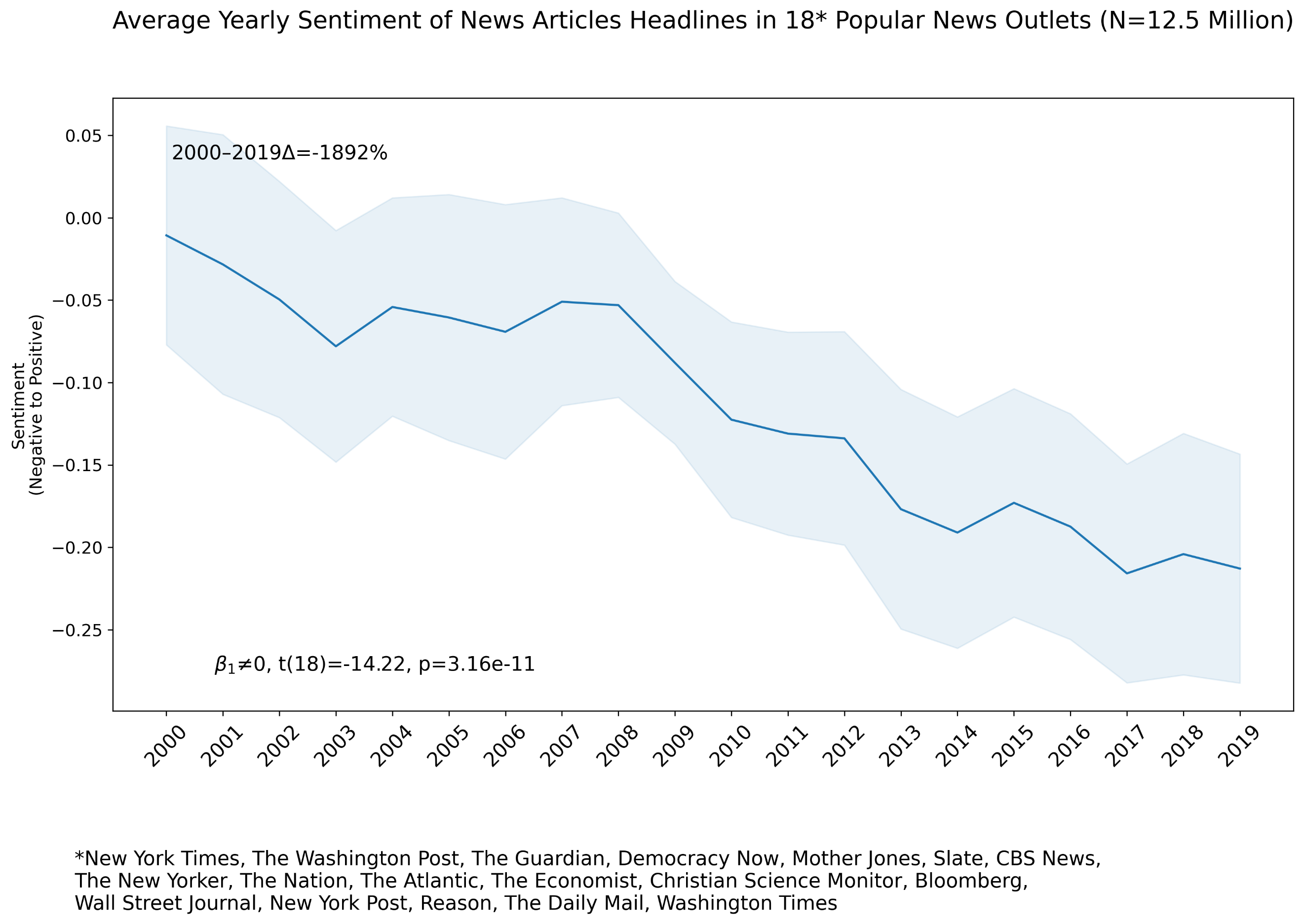


Figure S 13 The solid blue line shows the average yearly sentiment of headlines across 20 popular news media outlets using a DistilBert Transformer model fine-tuned on the SST-2 data set for sentiment annotations. The shaded gray area indicates the 95% confidence interval around the mean. A statistical test for the null hypothesis of zero slope is shown on the bottom left of the plot. The percentage change on average yearly sentiment across outlets between 2000 and 2019 is shown on the top left of the plot.

**Sentiment of news articles headlines by ideological leanings of news outlets using a fixed set of 18 news outlets with headlines availability since the year 2000**

The sentiment of news articles headlines from the 18 news outlets with online availability of headlines since the year 2000 is shown in Figure S 14. The pattern is similar to that shown in Figure 2 of the main manuscript that shows declining headlines sentiment for both left and right leaning news outlets. However, the effect size for right-leaning outlets is milder.


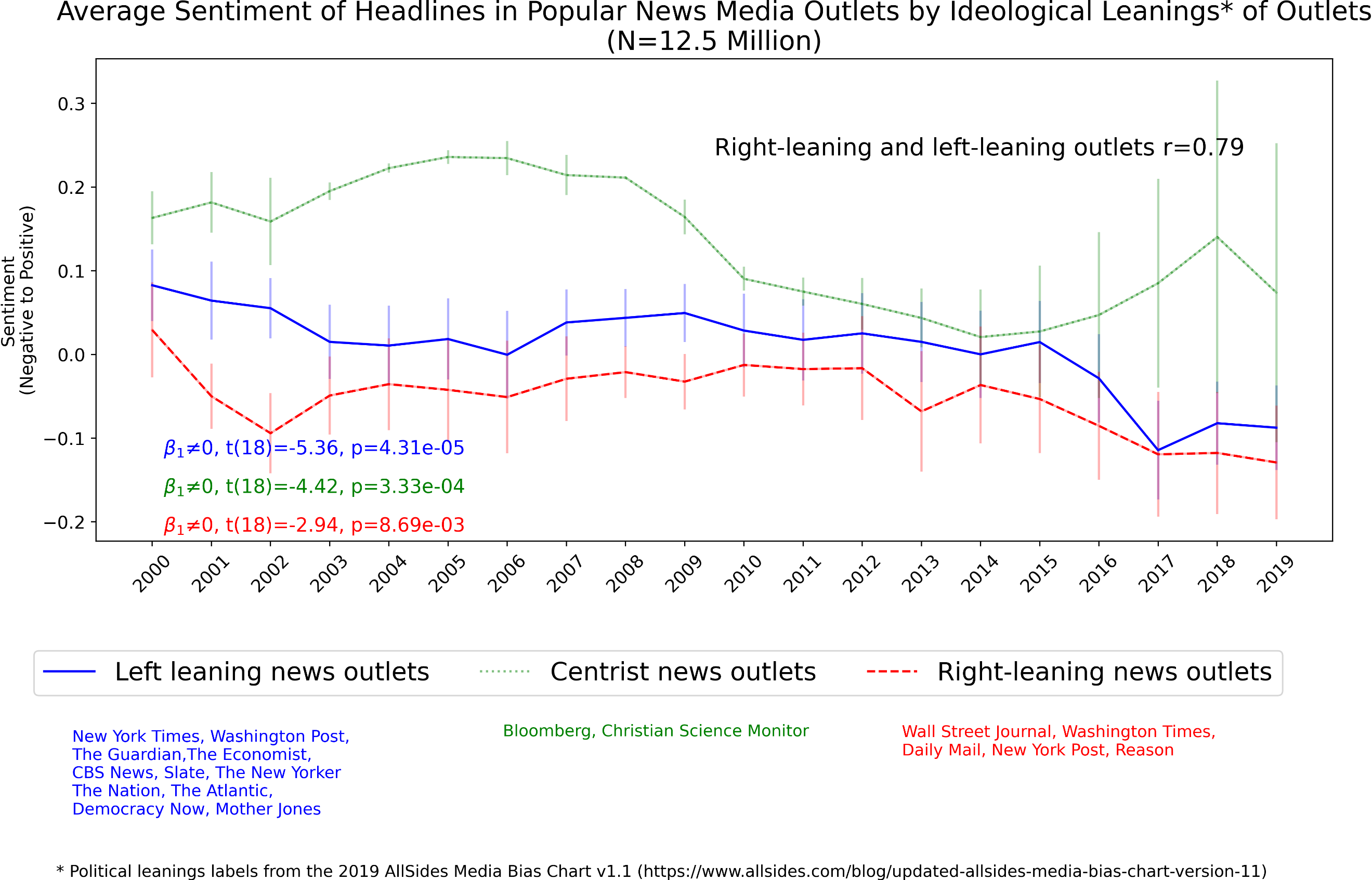


Figure S 14 Average yearly sentiment of headlines grouped by the ideological leanings of news outlets using human ratings of outlets political bias from the 2019 AllSides Media Bias Chart v1.1 [23]. The figure displays the standard error bars of the average yearly sentiment for outlets within each color-coded political orientation category. For each ideological grouping, statistical tests for the null hypothesis of zero slope are shown on the bottom left of the plot.

**Chronological analysis of emotion in news articles headlines using a fixed set of outlets with headlines availability since the year 2000**

The emotion of news articles headlines from news outlets with availability of headlines since the year 2000 is shown in Figure S 15. The pattern is similar to that of Figure 3 in the main manuscript.


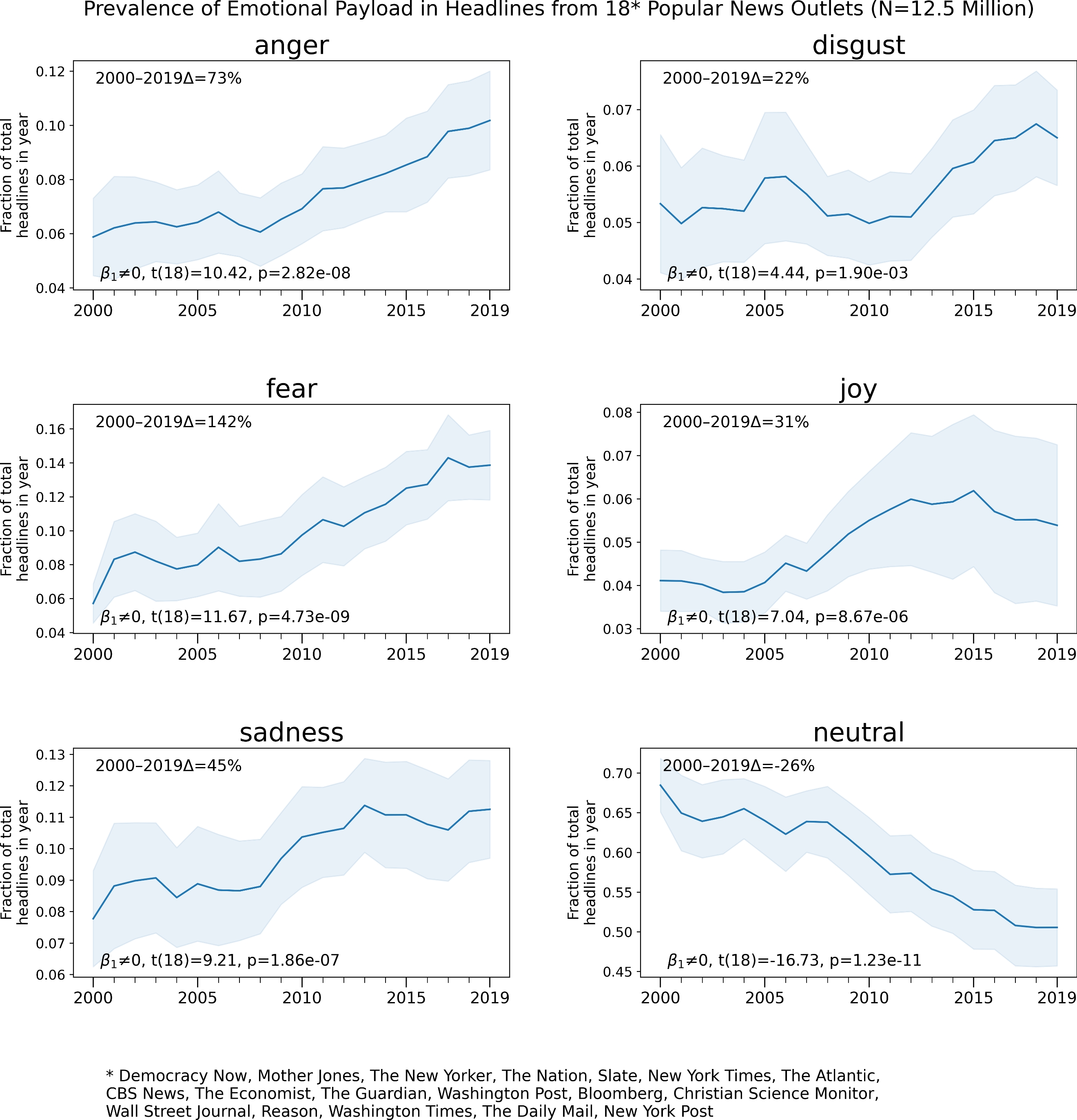


Figure S 15 Average yearly prevalence of news articles headlines denoting different types of emotionality in 18 popular news media outlets. The shaded gray area indicates the 95% confidence interval. Note the different scale of the Y axes for the different emotion types. For each emotional category, statistical tests for the null hypothesis of zero slope are shown on the bottom left of each subplot. Reported p-values have been Bonferroni-corrected for multiple comparisons. The percentage changes between 2000 and 2019 are shown on the top left of each subplot.

The emotion of news articles headlines from the 12 news outlets with availability of at least 2,000 headlines per year continuously since the year 2000 is shown in Figure S 16. The patterns in Figure S 16 are similar to those in Figure 3 of the main manuscript.


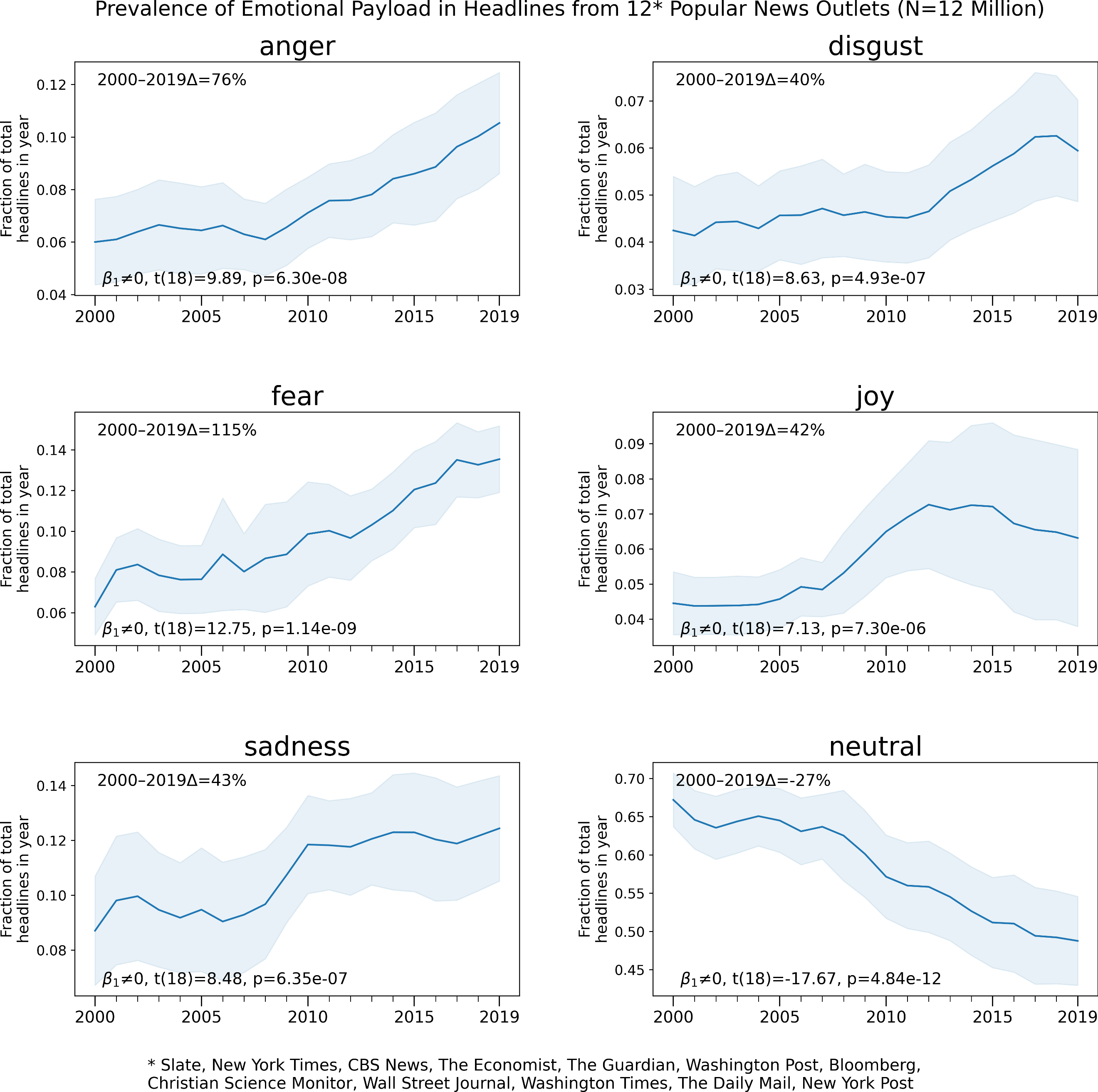


Figure S 16 Average yearly prevalence of news articles headlines denoting different types of emotionality in 12 popular news media outlets with at least 2,000 headlines per year since 2000. The shaded gray area indicates the 95% confidence interval. Note the different scale of the Y axes for the different emotion types. For each emotional category, statistical tests for the null hypothesis of zero slope are shown on the bottom left of each subplot. Reported p-values have been Bonferroni-corrected for multiple comparisons. The percentage changes between 2000 and 2019 are shown on the top left of each subplot.

The emotion of news articles headlines from the 6 news outlets with availability of at least 10,000 headlines per year continuously since the year 2000 is shown in Figure S 17. The patterns in Figure S 17 are similar to those in Figure 3 of the main manuscript.


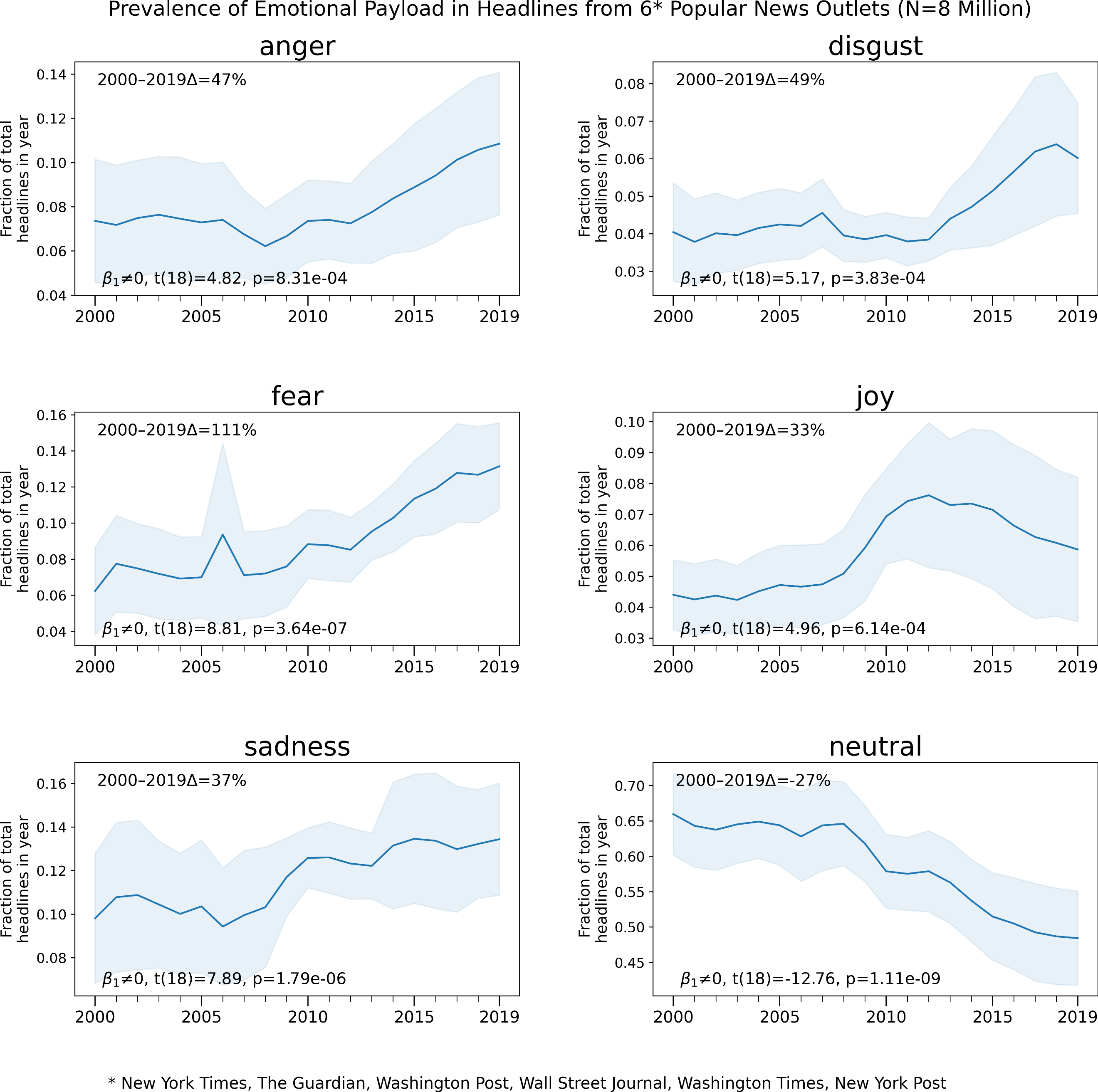


Figure S 17 Average yearly prevalence of news articles headlines denoting different types of emotionality in 6 popular news media outlets with at least 10,000 headlines per year since 2000. The shaded gray area indicates the 95% confidence interval. Note the different scale of the Y axes for the different emotion types. For each emotional category, statistical tests for the null hypothesis of zero slope are shown on the bottom left of each subplot. Reported p-values have been Bonferroni-corrected for multiple comparisons. The percentage changes between 2000 and 2019 are shown on the top left of each subplot.

**Emotion of news articles headlines by ideological leanings of news outlets using a fixed set of news outlets with headlines availability since the year 2000**

The emotionality of news articles headlines from the 18 news outlets with headlines availability since the year 2000 is shown in Figure S 18. The patterns are similar to those shown in Figure 4 of the main manuscript.


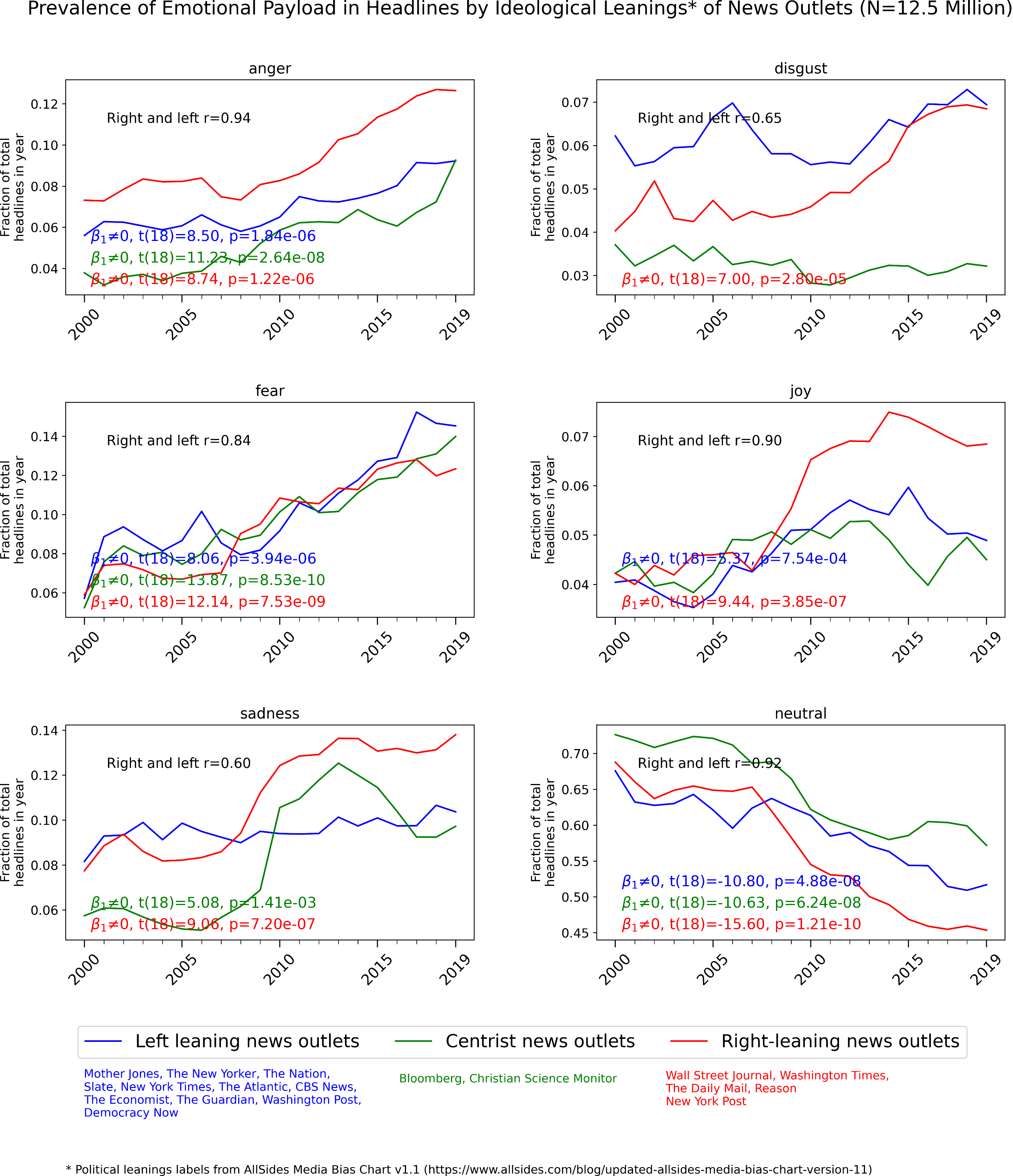


Figure S 18 Yearly prevalence of headlines denoting different types of emotionality in 18 popular news outlets grouped by human ratings of news media ideological orientation from the 2019 AllSides Media Bias Chart v1.1 [23]. Note the different scale of the Y axes for the different emotion types. Only statistical tests within each ideological grouping for which the null hypothesis of zero slope was rejected (after Bonferroni correction for multiple comparisons) are shown on the bottom left of each plot.

The emotionality of news articles headlines from the 12 news outlets with at least 2,000 headlines per year available since the year 2000 is shown in Figure S 19. The patterns are similar to those shown in Figure 4 of the main manuscript.


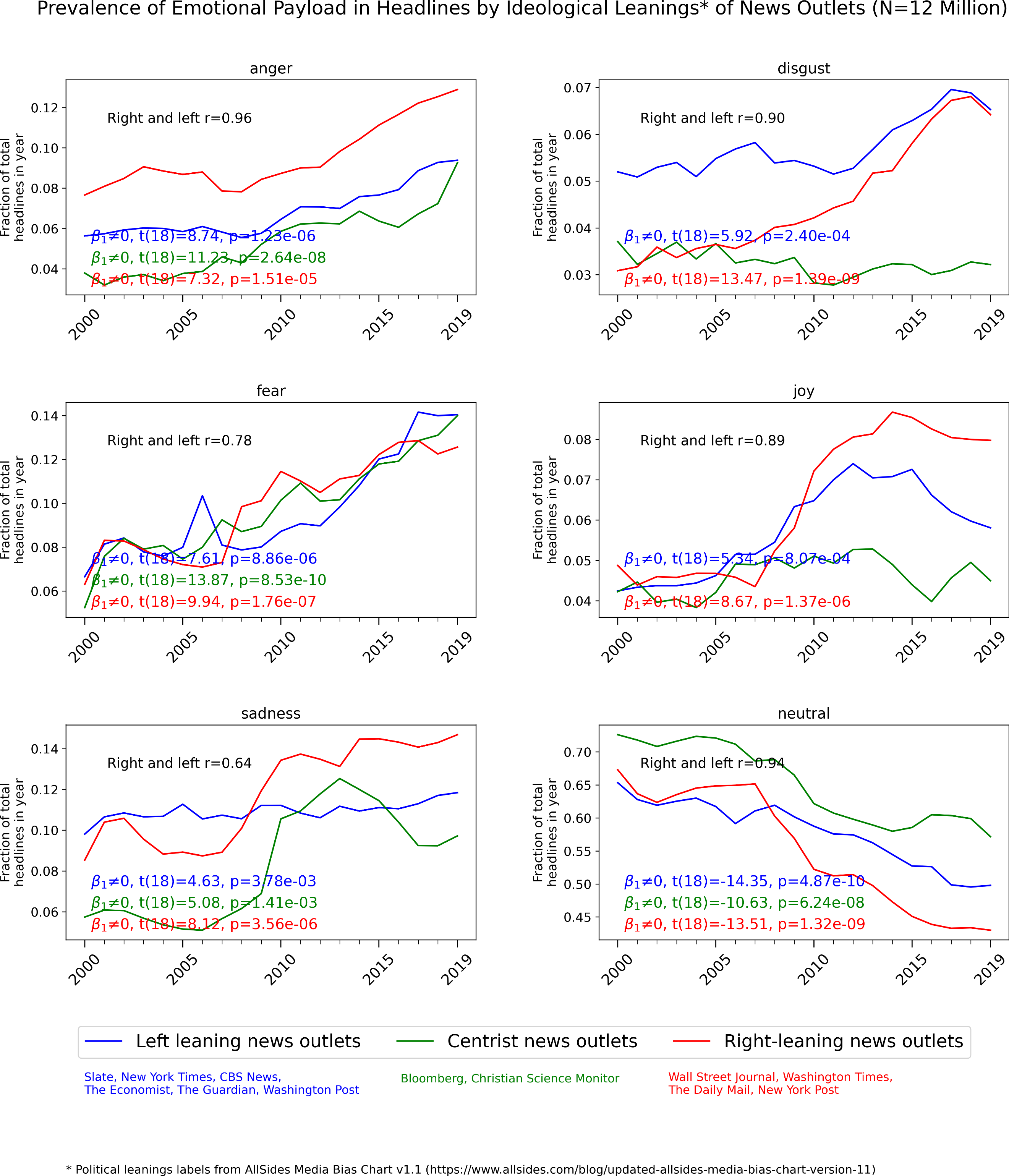


Figure S 19 Yearly prevalence of headlines denoting different types of emotionality in 12 popular news outlets grouped by human ratings of news media ideological orientation from the 2019 AllSides Media Bias Chart v1.1 [23]. Note the different scale of the Y axes for the different emotion types. Only statistical tests within each ideological grouping for which the null hypothesis of zero slope was rejected (after Bonferroni correction for multiple comparisons) are shown on the bottom left of each plot.

The emotionality of news articles headlines from the 6 news outlets with at least 10,000 headlines per year available since the year 2000 is shown in Figure S 20. The patterns are similar to those shown in Figure 4 of the main manuscript.


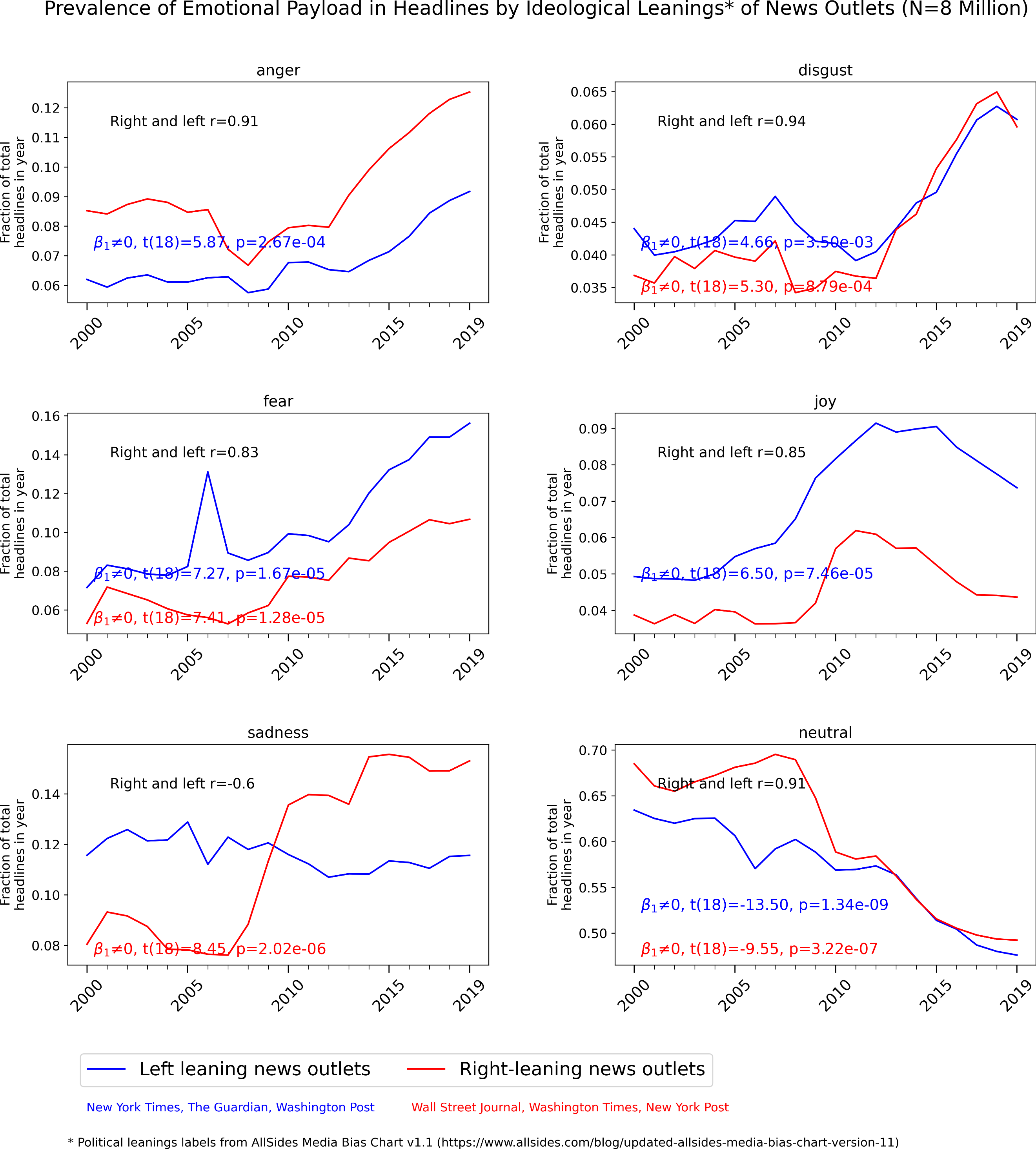


Figure S 20 Yearly prevalence of headlines denoting different types of emotionality in 6 popular news outlets grouped by human ratings of news media ideological orientation from the 2019 AllSides Media Bias Chart v1.1 [23]. Note the different scale of the Y axes for the different emotion types. Only statistical tests within each ideological grouping for which the null hypothesis of zero slope was rejected (after Bonferroni correction for multiple comparisons) are shown on the bottom left of each plot.

**Wordclouds**

As a sanity check, we visualize word clouds of 2019 headlines by model emotion prediction labels. We can observe in Figure S 21 how the terms *enjoy*, *celebrate*, *win*, *show*, *new* or *love* are prominent in headlines labelled by the model with the emotion of *joy*. In contrast, prominent words in headlines classified by the emotion model as displaying anger are: *accused*, *attack*, *protest*, *arrested*, *charged*, *murder*, *hit* or *death*. This provides further evidence about the validity of the automated emotion labelling annotation task.


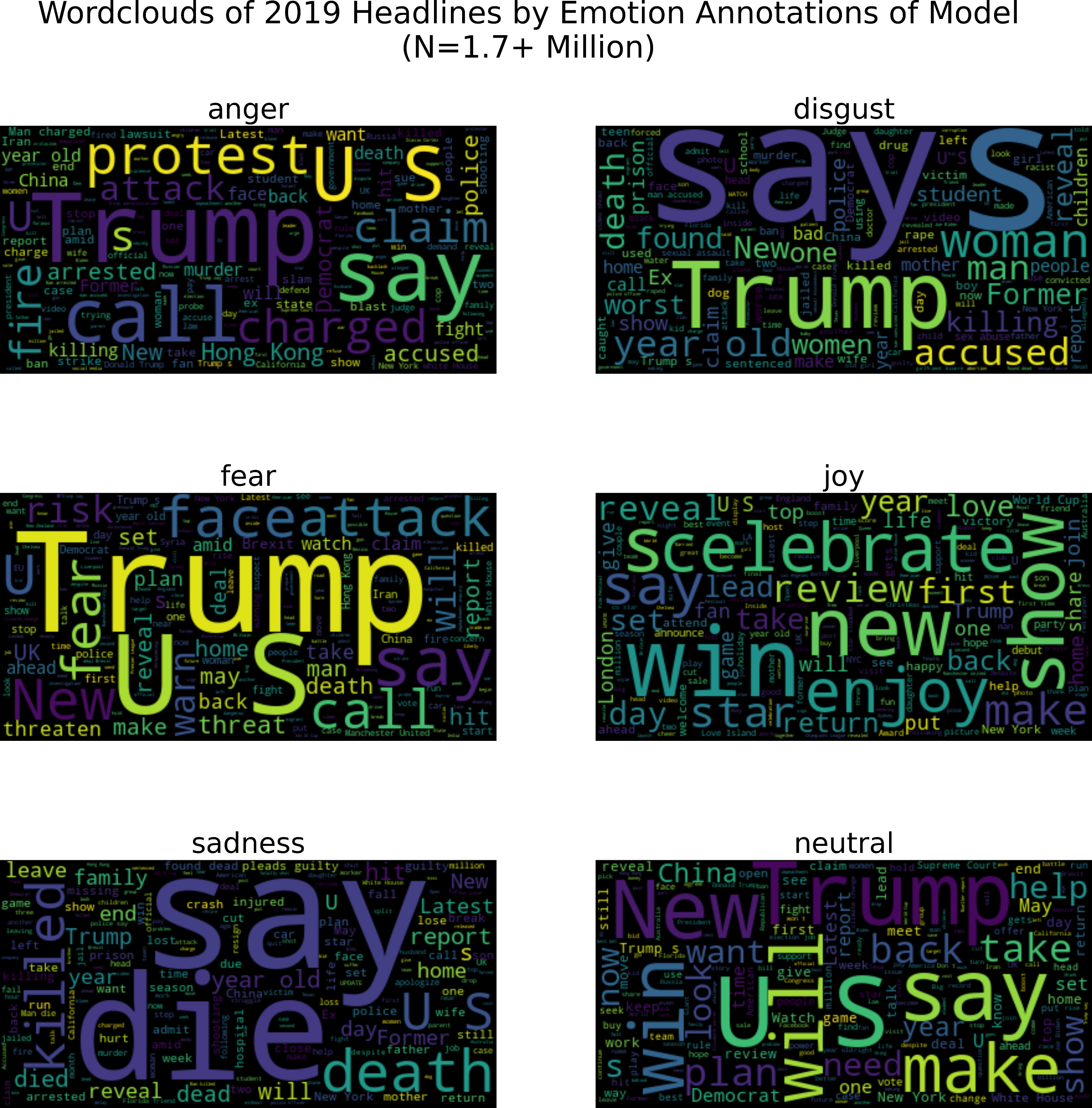


Figure S 21 Word clouds of 2019 headlines sorted by a DistilRoBERTa Transformer model emotion annotations.

**Models availability**

The SiEBERT Transformer model used for sentiment annotation and the DistilRoBERTa Transformer model fine-tuned on emotion labelling and used for emotion annotations in our analysis have been made publicly available by their original authors. Model weights, training hyper parameters and other relevant metadata about the models are available in the following repositories:

<https://huggingface.co/siebert/sentiment-roberta-large-english>

<https://huggingface.co/j-hartmann/emotion-english-distilroberta-base>

**Illustrative headlines sentiment annotations**

We provide in Table S 4 some illustrative sentiment annotations from New York Times 2019 headlines using the SiEBERT Transformer model for sentiment annotation:

| -0.9994863867759705 | Warning of ‘Fictitious’ Election Results Online, Congo Cuts Internet for 2nd Day |
| --- | --- |
| 0.9926884174346924 | Bright Spot for N.Y.’s Struggling Schools: Pre-K |
| 0.9985884428024292 | "The Best Movies and TV Shows New to Netflix, Amazon and More in January" |
| 0.997559666633606 | "Traffic Deaths in New York City Drop to 200, a Record Low" |
| -0.9987831711769104 | How Hollywood Gets the Publishing Industry Wrong |
| -0.9992159008979797 | Protests in Peru as Team Investigating Odebrecht Corruption Is Dismissed |
| -0.9994956851005554 | James Watson Had a Chance to Salvage His Reputation on Race. He Made Things Worse. |
| 0.9985467791557312 | How to Get Stronger in the New Year |

Table S 4 Illustrative sentiment annotations from New York Times 2019 headlines using the SiEBERT Transformer model.

**Illustrative emotion sentiment annotations**

We provide in Table S 5 some illustrative emotion annotations from Washington Post 2019 headlines using the DistilRoBERTa Transformer model for emotional annotation:

| joy | Watch NASA celebrate as New Horizons spacecraft completes most distant flyby |
| --- | --- |
| sadness | The Smithsonian and the National Gallery held on as long as they could. They’re closing. |
| disgust | These are the nastiest foods we tried in 2018 |
| anger | Trump’s latest assault on migrant children definitely won’t fly |
| fear | Dozens of tornadoes rip through Midwest |
| surprise | Surprise GOP tactical win in House exposes divisions in Democratic leadership |
| neutral | MIT builds backflipping four-legged robot cheetah |
| neutral | China plans another moon mission this year, eyes Mars in 2020 |

Table S 5 Illustrative emotion annotations from Washington Post year 2019 headlines using the DistilRoBERTa Transformer model.
